# Oxygen and ROS in Photosynthesis

**DOI:** 10.3390/plants9010091

**Published:** 2020-01-10

**Authors:** Sergey Khorobrykh, Vesa Havurinne, Heta Mattila, Esa Tyystjärvi

**Affiliations:** Department of Biochemistry/Molecular Plant Biology, University of Turku, FI-20014 Turku, Finland or khorobrykh@rambler.ru (S.K.); vetahav@utu.fi (V.H.); hkmatt@utu.fi (H.M.)

**Keywords:** reactive oxygen species, chloroplasts, photosynthetic electron transport chain, photodamage, redox signaling

## Abstract

Oxygen is a natural acceptor of electrons in the respiratory pathway of aerobic organisms and in many other biochemical reactions. Aerobic metabolism is always associated with the formation of reactive oxygen species (ROS). ROS may damage biomolecules but are also involved in regulatory functions of photosynthetic organisms. This review presents the main properties of ROS, the formation of ROS in the photosynthetic electron transport chain and in the stroma of chloroplasts, and ROS scavenging systems of thylakoid membrane and stroma. Effects of ROS on the photosynthetic apparatus and their roles in redox signaling are discussed.

## 1. Introduction

Molecular oxygen (O_2_) is a natural acceptor of electrons in the respiratory pathway of aerobic organisms and in many other biochemical reactions. In its ground state, O_2_ has two unpaired electrons with parallel spins in two separate π antibonding orbitals. Thus, ground-state O_2_ is a triplet diradical. According to Pauli’s principle, O_2_ reacts slowly with many biomolecules because spin restriction causes a kinetic barrier, as almost all biomolecules are in the singlet state, having paired electrons with opposite spins. The kinetic barrier of O_2_ is removed either by spin inversion of one unpaired electron or by a partial reduction in O_2_. Spin inversion requires the absorption of energy and converts the triplet state of O_2_ to the singlet state of molecular oxygen (^1^O_2_). All other forms of active oxygen are produced via an electron transfer mechanism. ^1^O_2_ and partially reduced forms of oxygen have a higher reactivity towards many organic molecules than the ground state of oxygen and they are collectively called reactive oxygen species (ROS).

ROS can be classified as radical and non-radical species. In addition, ROS can be roughly divided into free ROS, small molecules composed of oxygen and hydrogen only, and incorporated ROS, in which oxygen is bound to other molecules to form reactive oxygen derivatives. Furthermore, a family of reactive species containing nitrogen moieties associated with oxygen are classified as reactive nitrogen species, and reactive oxygen derivatives like lipid peroxyl radicals (LOO^•^) can be classified as both ROS and reactive lipid species. The free ROS are ^1^O_2_, superoxide anion radical (O_2_^•−^), hydroperoxyl radical (HO_2_^•^), hydrogen peroxide (H_2_O_2_), hydroxyl radical (HO^•^) and ozone (O_3_). Other molecules containing active oxygen are ROS derivatives. Table 1 presents the most important reactive species containing active oxygen according to this classification.

Chloroplasts convert light energy to energy for chemical bonds. Light absorption by the chlorophyll (Chl) molecules of Photosystems I and II (PSI and PSII) triggers a sequence of redox reactions along the thylakoid membrane. These reactions result in the oxidation of water, reduction of NADP^+^ to NADPH and formation of a proton gradient across the thylakoid membrane. In chloroplasts, O_2_ appears due to light-dependent water-splitting catalyzed by PSII. The steady-state concentration of O_2_ inside intact chloroplasts in the light depends on the external concentration of O_2_. At low external O_2_ concentrations (30 µM), the ratio of the internal to the external is about five, whereas at concentrations corresponding to those in air-saturated water (≈258 µM), the O_2_ concentration of isolated chloroplasts is similar to that of the medium [1].

^1^O_2_ is mainly formed via the interaction of O_2_ with the triplet excited state of chlorophyll (^3^Chl). The reduction in O_2_ by reduced forms of electron carriers in the photosynthetic electron transfer chain (PETC) can produce O_2_^•−^ and H_2_O_2_. The reaction of O_2_ with PETC is considered as the main source of ROS in chloroplasts, and light is essential for this ROS production. ROS can be interconverted by interaction with redox active compounds of the chloroplast, and ROS are produced both via O_2_^−^ mediated primary ROS generation mechanisms and ROS-mediated interconversion reactions.

ROS are unfavorable for chloroplast functions because ROS cause oxidative damage by reacting with biomolecules. Chloroplasts have ROS scavenging systems to prevent damage [2,3,4,5]. In addition to ROS-scavenging systems, chloroplasts have pathways, like non-photochemical quenching of excitation energy (NPQ), cyclic electron flow and plastid terminal oxidase (PTOX)-mediated chlororespiration, that diminish the appearance of long-lived redox active compounds [6,7,8]. All aerobic organisms have ROS-scavenging mechanisms to prevent ROS damage. Environmental stressors (high light, high or low temperature and others) enhance ROS production in chloroplasts and change the balance between ROS scavenging and ROS production. Imbalances between ROS production and scavenging cause changes in the redox state of the cell through a change in the levels of reduced and oxidized forms of antioxidants like ascorbate (AscH_2_), glutathione and thiol-containing compounds. Changes in the redox levels of the antioxidants trigger reactions where antioxidants act as ROS processing and signaling mediators that cause changes in gene expression [9,10]. In particular, the formation of ROS in Mehler’s reaction initiates light-signaling that depends on AscH_2_ and reduced glutathione (GSH) [11].

ROS production quenches excitation energy photochemically via the so-called water–water cycle that consists of the production of O_2_ in PSII, reduction in O_2_ to O_2_^•−^ and H_2_O_2_, scavenging of ROS, and regeneration of the scavenger [12]. The primary function of the water–water cycle is to scavenge ROS. Besides ROS scavenging and the photochemical quenching of excitation energy, the water–water cycle generates a proton gradient across the thylakoid membrane for both ATP production and the enhancement of NPQ [13].

Aerobic metabolism inevitably leads to the formation of ROS, and chloroplasts have to spend metabolic energy to build and maintain the ROS metabolism. The chloroplast provides light sensor functions for the whole plant cell, and the chloroplast ROS network participate in this signaling.

In the present review, we will mainly discuss the roles of ^1^O_2_, O_2_^•−^, HO_2_^•^, H_2_O_2_, HO^•^ and lipid peroxides in chloroplast metabolism. O_3_, although it is biologically important, is only shortly discussed because this ROS is not found within cells. The review presents the main properties of ROS and their typical reactions, the formation of ROS in the photosynthetic electron transport chain and in the stroma of chloroplasts, and the ROS-scavenging systems of thylakoid membrane and stroma.

## 2. ROS Properties and Basic Reactions

### 2.1. Singlet Oxygen, ^1^O_2_

Ground-state O_2_ is a triplet (^3^Σ^+^_g_O_2_) and can be converted to the singlet form via the absorption of energy that leads to spin inversion of one unpaired electron. Molecular oxygen has two singlet forms because two electrons with antiparallel spins may reside either in two different orbitals (^1^Σ^+^_g_O_2_) or both in one orbital (^1^∆gO_2_). The energy above the ground-state of O_2_ is 155 and 92 kJ/mol for ^1^Σ^+^_g_O_2_ and ^1^∆gO_2_, respectively [14]. ^1^Σ^+^_g_O_2_ is rapidly converted to ^1^∆gO_2_ or ^3^Σ^+^_g_O_2_ and, in the liquid phase, the lifetime of ^1^Σ^+^_g_O_2_ is only 10^−11^ s [14], which is too short for ^1^Σ^+^_g_O_2_ to take part in biochemical reactions. Therefore, ^1^O_2_ will be used here to designate ^1^∆gO_2_. O_2_ will be used to designate ^3^Σ^+^_g_O_2_.

#### 2.1.1. Formation of ^1^O_2_

The most common mechanism of ^1^O_2_ generation is photosensitization, i.e., the reaction of O_2_ with a photoexcited sensitizer dye (S*). Both forms of ^1^O_2_, ^1^Σ^+^_g_O_2_ and ^1^∆gO_2_, can be produced via the spin-conserved Reactions (1) and (2).

^1^S* + O_2_ → ^3^S + ^1^O_2_ (1)

^3^S + O_2_ → ^1^S + ^1^O_2_ (2)

The second reaction is more common because singlet excited states (^1^S*) are usually short-lived and because only a few dye molecules have a large enough energy gap between the ^1^S* and triplet states (^3^S) to convert O_2_ to ^1^O_2_ [15]. ^3^Chl reacts rapidly with O_2_ with a second-order rate constant close to 10^9^ M^−1^ s^−1^, and the relative quantum yield of ^1^O_2_ generation by chlorophyll *a* (Chl *a*) was around 80% when meso-tetraphenylporphyrin and tetra(*p*-sulfophenyl) porphyrin were used as standards [16]. The spin transition O_2_ → ^1^Σ^+^_g_O_2_ is associated with the absorption band of gaseous O_2_ at 762 nm. Absorption at 1268 nm, in turn, was found for the transition O_2_ → ^1^∆gO_2_ in liquids and in the atmosphere [17].

In addition to photosensitized generation, ^1^O_2_ can be produced by several chemical reactions that usually involve reduced forms of oxygen like H_2_O_2_, O_2_^•−^ and reactive oxygen derivatives like organic peroxides (ROOH) and peroxyl radicals (ROO^•^) [14,18].

^1^O_2_ is produced by decomposition of H_2_O_2_ via the Haber–Weiss mechanism (Reactions (3) and (4)) [19,20].

H_2_O_2_ + O_2_^•−^ → HO^•^ + HO^−^ + (O_2_ or ^1^O_2_) (3)

H_2_O_2_ + HO_2_^•^ → HO^•^ + H_2_O + (O_2_ or ^1^O_2_) (4)

The rate constants of Reactions (3) and (4) in aqueous medium are 0.13 to 0.23 M^−1^ s^−1^ and 0.5 M^−1^ s^−1^, respectively [21,22,23]. It has been suggested that the biologically important oxidant produced by Reaction (3) is HO^•^ rather than ^1^O_2_ [24]. ^1^O_2_ can also be produced by O_2_^•−^ dismutation (Reaction (5)) or by electron transfer from O_2_^•−^ to radical (A^•^) or non-radical (A) electron acceptors (Reactions (6) and (7)) [25,26,27].

O_2_^•−^ + O_2_^•−^ + 2H^+^ → H_2_O_2_ + (O_2_ or ^1^O_2_) (5)

O_2_^•−^ + A^+^ → (O_2_ or ^1^O_2_) + A^•^ or O_2_^•−^ + A^•+^ → (O_2_ or ^1^O_2_) + A (6)

O_2_^•−^ + HO^•^ → HO^−^ + ^1^O_2_ (7)

Efficient production of ^1^O_2_ was found in a reaction of O_2_^•−^ with benzoyl peroxide or lauroyl peroxide (Reaction (8)) [28].

2O_2_^•−^ + RCOOCR → 2RCO_2_^−^ +2^1^O_2_ (8)

^1^O_2_ can be generated via the Russel mechanism (Reaction (9)), in which two ROO^•^ radicals react to form a linear tetraoxide (ROOOOR) intermediate that rapidly decomposes to the corresponding ketone (R=O), alcohol (R–OH) and ^1^O_2_ [29]. In fact, ROOOOR decomposition releases either ^1^O_2_ or excited triplet carbonyl (R=^3^O*). However, the relative yield of ^1^O_2_ is 10%, while the relative yield of R=^3^O* is only 0.01% [30,31,32].

ROO^•^ + ROO^•^ ∆ ROOOOR → R–OH + R=O + ^1^O_2_ (9)

^1^O_2_ can be produced in the reaction of O_2_ with an R=^3^O* (Reaction (10)), which can be formed by the decomposition of ROOOOR (Reaction (9)) [33].

R=^3^O* + O_2_ → R=O + ^1^O_2_ (10)

Other specific ions, like the hypohalite ion OCl^−^ and the molybdate ion MoO_4_^2−^, can react with H_2_O_2_, forming ^1^O_2_ (Reactions (11)–(13)) [34,35,36]; these reactions are probably not of biological importance.

H_2_O_2_ + H^+^ + OCl^−^ → H_2_O + HCl + ^1^O_2_ (11)

MoO_4_^2−^ + H_2_O_2_ ∆ MoO_6_^2−^ + 2H_2_O (12)

MoO_6_^2−^ → MoO_4_^2−^ + ^1^O_2_ (13)

#### 2.1.2. Physical Deactivation of ^1^O_2_

Both excited singlet states of oxygen are metastable and can lose excitation energy via radiative and non-radiative pathways. The latter is physical quenching of ^1^O_2_. The radiative deactivation is the transition of ^1^∆gO_2_ to O_2_ associated with light emission (hν; Reaction (14)).

^1^O_2_ → O_2_ + *h*ν (14)

The phosphorescence spectrum has a major maximum at 1268 nm [37]. The phosphorescence is extremely weak, as the deactivation of ^1^O_2_ mostly proceeds non-radiatively due to the collision of ^1^O_2_ with another molecule. The quantum yield of luminescence is from 10^−6^ to 10^−3^ [32]. Non-radiative deactivation mechanisms include electronical-to-vibrational energy transfer, charge-transfer-induced quenching and electronic energy transfer. In the deactivation of ^1^Σ^+^gO_2_ and ^1^∆gO_2_ by an electronic-to-vibrational process, the excitation energy of ^1^O_2_ is converted into vibration of the O_2_ molecule and a quencher molecule Qr (Reaction (15)).

^1^O_2_ (*v* = 0) + Qr (*v* = 0)→ O_2_ (*v* = m) + Qr (*v* = n) + E_mn_ (15)

where E_mn_ is the energy difference between the reactants and the products and *v* is the vibrational energy level of a molecule; m and n are vibrational modes.

The deactivation of ^1^O_2_ by collisions of ^1^O_2_ with other molecules limits the lifetime of ^1^O_2_ in many solvents. The lifetime of ^1^O_2_ for many organic solvents is within 8–100 µs. The substitution of hydrogen with deuterium in the solvent molecule leads to a significant increase in the lifetime of ^1^O_2_, usually by a factor of ten or more [17,38,39,40]. The second order rate constant for the deactivation of ^1^O_2_ via an electronic-to-vibrational process varies widely, from 10^−2^ to 10^6^ M^−1^ s^−1^ [17].

In addition to the electronic–vibrational non-radiative deactivation, ^1^O_2_ can be deactivated via charge-transfer-induced quenching (Reaction (16)) and an electronic energy transfer mechanism (Reaction (17)).

^1^O_2_ + ^1^A ∆ ^1^(O_2_ A) ∆ ^3^(O_2_ A) → O_2_ + ^1^A (16)

^1^O_2_ + A → ^3^(O_2_ A) → O_2_ + ^3^A, (17)

where A is an acceptor.

Molecules with high triplet energies (more than 94 kJ mol^−1^) and low oxidation potential (midpoint redox potential (*E_m_*) around 1.9 V vs. Normal Hydrogen Electrode (NHE)) can deactivate ^1^O_2_ with the charge-transfer mechanism. Second-order rate constants for deactivation of ^1^O_2_ via the charge-transfer mechanism are within 10^3^ to 10^9^ M^−1^ s^−1^ [17]. In the charge-transfer mechanism, the ^1^(O_2_ A) complex finally dissociates to A and O_2_ without charge separation. Electronic energy-transfer quenching of ^1^O_2_ occurs via the interaction of molecules with a lower triplet state energy than the energy of ^1^O_2_. The deactivation of ^1^O_2_ via the electronic energy-transfer mechanism is very efficient and its second-order rate constant is close to the diffusion-controlled limit. Carotenoids including β-carotene and lutein are the most efficient quenchers of ^1^O_2_, and the second order rate constant for many carotenoids is about 10^10^ M^−1^ s^−1^ [17,41].

#### 2.1.3. Chemical Reactions of ^1^O_2_

The term “chemical deactivation” of ^1^O_2_ can be applied to reactions in which the products have less reactivity and toxicity towards cell metabolism than ^1^O_2_. The redox potential relative to NHE for the pair ^1^O_2_/O_2_^•−^ is 0.65 V [42]. ^1^O_2_ is an electrophilic agent and reacts with electron-rich organic molecules via three well-known mechanisms.

The ene reaction (Alder-ene reaction) is associated with the formation of a hydroperoxide (Reaction (18)).

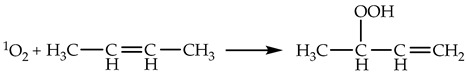
(18)

Cycloaddition is associated with dioxetane formation (Reaction (19)).

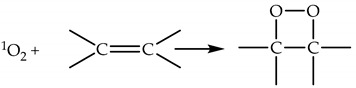
(19)

Cycloaddition is associated with aromatic compounds and formation of an endoperoxide via the Diels–Alder mechanism (Reaction (20)).

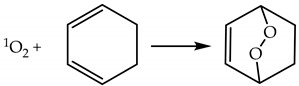
(20)

^1^O_2_ can react with the unsaturated fatty acids of membrane lipids to form both conjugated and non-conjugated diene hydroperoxides (Reaction (21)) with a second order rate constant of about 10^4^ M^−1^ s^−1^ [4,43].

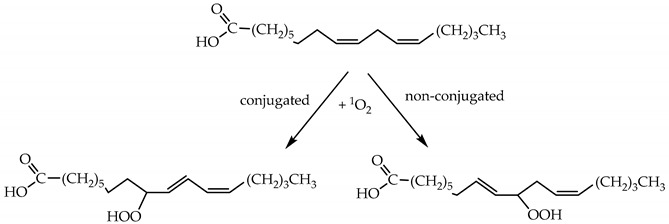
(21)

^1^O_2_ can react with amino acids that have double bonds or an electron-rich sulfur atom, such as tryptophan, histidine, tyrosine, methionine and cysteine, to form corresponding peroxides. The second-order rate constant for reaction of ^1^O_2_ with those amino acids is around 10^7^ M^−1^ s^−1^ [44]

^1^O_2_ can efficiently oxidize amines to imines with the formation of HO_2_^•^ (Reaction (22)) [45].

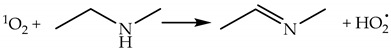
(22)

^1^O_2_ can oxidize electron-rich compounds like phenols to benzoquinones by an electron transfer mechanism (Reaction (23)) [46].

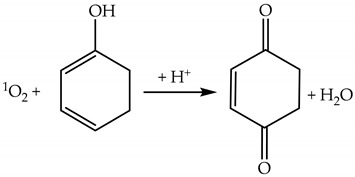
(23)

The oxidation of AscH_2_ (Reaction (24)) and plastoquinol (PQH_2_) (Reaction (25)) by ^1^O_2_ can proceed as a two-electron reduction of ^1^O_2_ to H_2_O_2_ [47,48].

AscH_2_ + ^1^O_2_ → DHA + H_2_O_2_ (24)

PQH_2_ + ^1^O_2_ → PQ + H_2_O_2_ (25)

where DHA and PQ are dehydroascorbate and plastoquinone, respectively.

The second-order rate constant for the reaction of ^1^O_2_ with AscH_2_ depends on pH and varies from 10^5^ M^−1^ s^−1^ to 10^8^ M^−1^ s^−1^ [49]. Prenyllipids like PQH_2_-9 and α-tocopherol react with ^1^O_2_ with second-order rate constants of about 10^8^ M^−1^ s^−1^ [50].

#### 2.1.4. Lifetime and Diffusion Distance of ^1^O_2_

Solvents and other deactivating compounds play significant roles in controlling the lifetime of ^1^O_2_ and the lifetime, in turn, determines both the diffusion distance and the ability of ^1^O_2_ to react with other substances. The lifetimes of ^1^O_2_ in a pure lipid membrane and in the thylakoid membrane have been estimated to be 7 µs and 70 ns, respectively [51]. Thus, the respective diffusion distances, approximated using the diffusion coefficient of O_2_, are 220 and 5.5 nm. The very short lifetime in the thylakoid membrane may be caused by a high concentration of compounds deactivating ^1^O_2_. In a nerve cell, the lifetime of ^1^O_2_ is about 200 ns, which leads to a diffusion distance of about 270 nm [52]. The lifetimes and diffusion distances of ^1^O_2_ in different tissues have been recently reviewed [23].

### 2.2. Superoxide Anion Radical, O_2_^•−^

The O_2_^•−^ is the one-electron reduced form of molecular oxygen. Detailed analyses of the properties and biological roles of O_2_^•−^ are available in several comprehensive reviews [53,54,55,56]. O_2_^•−^ is a deprotonation agent and can be protonated to HO_2_^•^ (Reaction (26)).

O_2_^•−^ + H^+^ → HO_2_ (26)

The deprotonation ability of O_2_^•−^ depends strongly on the solvent. In aqueous solutions, O_2_^•−^ is a weak deprotonation agent with pKa of 4.8 due to strong solvation of O_2_^•−^. The free energy of hydration for O_2_^•−^ was estimated to be around 355 kJ mol^−1^ [57]. Therefore, only 0.25% of O_2_^•−^ is protonated at physiological pH values. In non-aqueous solutions like organic solvents, O_2_^•−^ is a strong deprotonation agent. For example, in dimethylformamide (DMF), the pKa value of HO_2_^•^ is around 12 [54]. However, in reality the protonation of O_2_^•−^ in the presence of a protonated compound AH can accompany the reduction of HO_2_^•^ to hydroperoxyl anion (HO_2_^−^) and occur via a two-step mechanism (Reactions (27) and (28)), which makes O_2_^•−^ a much stronger deprotonation agent than would follow from its basicity.

O_2_^•−^ + AH ∆ HO_2_^•^ + A^−^ (27)

O_2_^•−^ + HO_2_^•^ → O_2_ + HO_2_^−^ (28)

which sum up to

2O_2_^•−^ + AH ∆ O_2_ + HO_2_^−^ + A^−^. (29)

The equilibrium constant of Reaction (29) is about 10^9^ [54]. Therefore, in the deprotonation process the pKa value of O_2_^•−^ should be considered equivalent to a base with pKa 24 [53,54]. The *E_m_* of the pair O_2_/O_2_^•−^ is pH-dependent, due to protonation of O_2_^•−^ and formation of HO_2_^•^. In an aqueous solution at pH 7, the *E_m_* of the pair O_2_/O_2_^•−^ is −160–−180 mV vs. NHE, and the *E_m_* value becomes more positive under a low pH, around 100 mV [12,58]. In aprotic media offering only a weak solvation of O_2_^•−^, O_2_^•−^ acts as a strong reductant and the redox potential of the pair O_2_/O_2_^•−^ is estimated to range between −550 and −600 mV vs. NHE [54] in DMF and around −640 mV in acetonitrile [59,60].

#### 2.2.1. Formation of O_2_^•−^

O_2_^•−^ is mainly formed via the interaction of O_2_ with reduced compounds having a low redox potential (A) (Reaction (30)).

A^−^ + O_2_ ∆ A + O_2_^•−^ (30)

O_2_^•−^ can be formed in a potentially important equilibrium reaction with semiquinone anion radicals (Q^•−^) with the formation of the respective quinone Q (Reaction (31)).

Q^•−^ + O_2_ ∆ Q + O_2_^•−^ (31)

The equilibrium constant of Reaction (31) can be determined from the redox potentials of Q/Q^•−^ and O_2_/O_2_^•−^. In aqueous solutions at pH 7, the equilibrium constant for Reaction (31) is estimated as 2 × 10^−5^ for benzosemiquinone with a redox potential around 100 mV, and 26 for durosemiquinone with redox potential around −260 mV [61]. The formation of O_2_^•−^ via Reaction (31) is favorable for Q^•−^, with the redox potential of the Q/Q^•−^ pair lower than 160–−180 mV because, with this redox potential, the forward rate constant (*k_forward_*) of Reaction (31) is larger than the reverse rate constant (*k_reverse_*). Reaction (31) when Q^•−^ is benzosemiquinone proceeds with *k_forward_* of 5 × 10^5^ M^−1^ s^−1^ and with *k_reverse_* of 10^8^ M^−1^ s^−1^. For durosemiquinone, *k_forward_* and *k_reverse_* were estimated to be 2.2 × 10^8^ M^−1^ s^−1^ and 10^7^ M^−1^ s^−1^, respectively [61]. However, if O_2_^•−^ is efficiently removed after Reaction (31), then the rate of formation depends only on *k_forward_*.

#### 2.2.2. Reactions of O_2_^•−^

Reactions of O_2_^•−^ with organic and inorganic molecules can proceed in five ways: deprotonation reaction (protonation of O_2_^•−^ by H^+^ or the attraction of a proton from a proton donor), attraction of hydrogen, electron transfer reaction, nucleophilic substitution or addition, and addition to a metal or metal complex.

In electron transfer reactions, O_2_^•−^ can act as both an oxidant and a reductant. In many cases, the electron transfer reaction involves a deprotonation reaction (deprotonation–oxidation mechanism). O_2_^•−^ can reduce or oxidize organic and inorganic molecules like quinones and cytochromes (cyt) or transition metal ions in equilibrium reactions via a one-electron transfer mechanism, Reactions (31)–(33).

O_2_^•−^ + cyt (Fe^3+^) ∆ O_2_ + cyt (Fe^2+^) (32)

O_2_^•−^ + Fe^3+^ ∆ O_2_ + Fe^2+^ (33)

In many cases, O_2_^•−^ oxidizes organic and inorganic molecules by hydrogen attraction via the deprotonation–oxidation mechanism. Oxidation of AscH_2_ by O_2_^•−^ in aqueous solution (Reaction (34)) proceeds as a two-step reaction (Reactions (35) and (36)) with a second-order rate constant of 3.3 × 10^5^ M^−1^ s^−1^ at pH 7.8 [62,63,64].

O_2_^•−^ + AscH_2_ → H_2_O_2_ + Asc^•^^−^ (34)

O_2_^•−^ + AscH_2_ ∆ HO_2_^•^ + AscH^−^ (35)

HO_2_^•^ + AscH^−^ → H_2_O_2_ + Asc^•−^ (36)

The same mechanism is suggested for oxidation by O_2_^•−^ of thiols as in reduced GSH and lipophilic compounds as in α-tocopherol, Reactions (37)–(40), respectively [64,65].

O_2_^•−^ + GSH ∆ + HO_2_^•^ + GS^−^ (37)

HO_2_^•^ + GS^−^ ∆ + HO_2_^−^ + GS^•^ (38)

O_2_^•−^ + α-Tocopherol-H ∆ HO_2_^•^ + α-Tocopherol^−^ (39)

HO_2_^•^ + α-Tocopherol^−^ ∆ HO_2_^−^ + α-Tocopherol (40)

In Reactions (37) and (38), GS^−^ and GS^•^ stand for deprotonated reduced glutathione and oxidized glutathione, respectively.

The second-order rate constant of the reaction of O_2_^•−^ with α-tocopherol incorporated into soybean or dimyristoyl phosphatidylcholine liposomal membranes was estimated to be 4.9 × 10^3^ M^−1^ s^−1^ [62]. The second-order rate constant of the reaction of O_2_^•−^ with GSH was estimated to be 10^3^ M^−1^ s^−1^ [66,67].

The main mechanism of deactivation of O_2_^•−^ is spontaneous or enzymatic dismutation (Reaction (5)). Non-enzymatic dismutation of O_2_^•−^ usually proceeds in aqueous solution and very strongly depends on pH because the protonation of O_2_^•−^ determines the rate. Dismutation can be considered as a two-step reaction: protonation of O_2_^•−^, Reaction (41) and a radical–radical reaction between O_2_^•−^ and HO_2_^•^ or between two molecules of HO_2_^•^—Reactions (42) and (43), respectively.

O_2_^•−^ + H^+^ ∆ HO_2_^•^, pKa = 4.8 (41)

O_2_^•−^ + HO_2_^•^ + H^+^→ H_2_O_2_ + O_2_ (42)

HO_2_^•^ + HO_2_^•^ → H_2_O_2_ + O_2_ (43)

The second-order rate constant of the dismutation of O_2_^•−^ has a maximum (10^8^ M^−1^ s^−1^) at pH 4.8, equal to the pKa value of HO_2_^•^. The rate constant decreases with increasing pH and becomes very low, around 0.3 M^−1^ s^−1^, at alkaline pH. At physiological pH, the rate constant is about 10^5^ M^−1^ s^−1^ [68]. The enzymatic dismutation of O_2_^•−^ is catalyzed by superoxide dismutase (SOD, EC 1.15.1.1). The SOD-catalyzed reaction proceeds as a sequence of oxidation and reduction of O_2_^•−^ by a metal ion (M) of the SOD enzyme, Reactions (44) and (45).

M^(n+1)+^-SOD + O_2_^•−^ → M^n+^-SOD + O_2_ (44)

M^n+^-SOD + O_2_^•−^ + 2H^+^ → M^(n+1)+^-SOD + H_2_O_2_ (45)

The rate constant of O_2_^•−^ dismutation catalyzed by SOD is about 6.4 × 10^9^ M^−1^ s^−1^ [69].

O_2_^•−^ is a powerful nucleophile in aprotic medium and can be involved in nucleophilic reactions with various organic compounds. O_2_^•−^ reacts with alkyl halides (RX), acyl halides and acyl anhydrides to form ROO^•^ intermediates through nucleophilic substitution reactions [54]. O_2_^•−^ can add to positively charged carbon–carbon double bonds [70] and carbon–nitrogen double bonds [71]. For example, Reactions (46)–(48) illustrate the reaction of O_2_^•−^ with RX, acyl halide and anhydride, respectively. The peroxy and alkoxy radicals are more reactive than O_2_^•−^ itself.

O_2_^•−^ + RX → ROO^•^ + X^−^ (46)

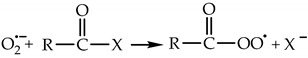
(47)

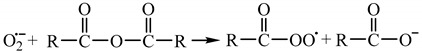
(48)

In addition to nucleophilic reactions with organic molecules, O_2_^•−^ can bind to both transition metals and to metal complexes. For example, in PSII, the interaction of O_2_^•−^ with a ferrous heme iron leads to the formation of a ferric–peroxo ((Fe^3+^)-OO^−^) complex which can be protonated to a ferric–hydroperoxo ((Fe^3+^)-OOH) complex, Reaction (49) and (50) [72].

O_2_^•−^ + L-(Fe^2+^) → L-(Fe^3+^)-OO^−^ + H^+^ (49)

L-(Fe^3+^)-OO^−^ + H^+^ → L-(Fe^3+^)-OOH (50)

where L is a ligand.

#### 2.2.3. Lifetime and Diffusion Distance of O_2_^•−^

The lifetime of O_2_^•−^ is controlled by dismutation (Reaction (5)). Thus, in the absence of SOD, the lifetime of O_2_^•−^ depends on pH in aqueous solutions and on the presence of a proton donor in aprotic media. O_2_^•−^ is more stable in alkaline aqueous solutions (*t*_1/2_ = 50 s at pH 14), and the lifetime of O_2_^•−^ decreases with decreasing pH (*t*_1/2_ = 0.2 s at pH 10). The lifetime of O_2_^•−^ prepared in a two-electrode cell in DMF was found to be 76 min at 0 °C for 0.1 M O_2_^•−^, and around 35 h for the O_2_^•−^ concentrations from 0.001 to 0.01 M [54]. In cells, the lifetime of O_2_^•−^ is efficiently controlled by SOD, and the lifetime will depend on SOD activity. In the periplasm of *Escherichia coli*, the lifetime of O_2_^•−^ was estimated to be longer than 0.6 s using the rate of O_2_^•−^ formation and the rate constant of its dismutation. The diffusion distance was calculated as 35 µm, assuming a general diffusion coefficient of small molecules of about 10^5^ cm^2^ s^−1^ [73].

### 2.3. Hydrogen Peroxide, H_2_O_2_

H_2_O_2_ is the result of two-electron reduction of O_2_ and considered a major biological ROS. In cells, H_2_O_2_ is mostly present in the neutral form because its pKa is 11.8. H_2_O_2_ is a strong, two-electron oxidant with a standard redox potential (*E*_0_*′*) of 1.32 V at pH 7.0. However, H_2_O_2_ reacts slowly or does not react with most biological molecules, including low-molecular-weight antioxidants, due to a high activation energy barrier [74]. Even if a reaction with H_2_O_2_ is thermodynamically favorable, it may be very slow.

Low-potential compounds reduce H_2_O_2_ with one electron, as the redox potential of H_2_O_2_/HO^•^ is 0.3 V [12]. H_2_O_2_ can also act as an electrophile due to the polarizability of the O–O bond. H_2_O_2_ has a permanent dipole moment of 2.26 Debye. The O–O bond is relatively weak and susceptible to homolysis. H_2_O_2_ is decomposed by heating, radiolysis, photolysis, or by reaction with redox active transition metals [74].

#### 2.3.1. Formation of H_2_O_2_.

Reduction of O_2_ to O_2_^•−^ followed by its dismutation (Reaction (5)) is the main pathway for the formation of H_2_O_2_ in cells.

H_2_O_2_ can be formed via oxidation of a quinol by O_2_. For example, hydroanthraquinone is widely used for the commercial synthesis of H_2_O_2_, Reaction (51) [75].

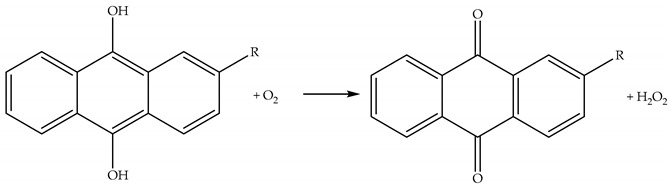
(51)

H_2_O_2_ can be produced by a reaction of ^1^O_2_ or O_2_^•−^ with an electron donor, like AscH_2_, Reactions (24) and (34), respectively [47,64].

The main reaction of ^1^O_2_ with PQH_2_ in methanol was found to result in the formation of PQ and H_2_O_2_, Reaction (25) and the amount of H_2_O_2_ produced was essentially the same as the amount of oxidized PQH_2_ [48].

The firect formation of H_2_O_2_ in a reaction of O_2_ and H_2_ can occur over catalysts containing palladium (PdAu, Pd-SiO_2_, PdZn and others), Reaction (52) [76].

(52) $$O_{2}+H_{2}\;\underset{\to }{\mathrm{Pd-catalyst}}\;H_{2}O_{2}$$

No direct formation of H_2_O_2_ from H_2_ and O_2_ is expected in aerobic cells because the production of hydrogen requires anaerobic conditions [77]. H_2_ is consumed by the bidirectional hydrogenase in green algae [78], but an enzyme-catalyzing Reaction (52) has not been found.

#### 2.3.2. Reactions of H_2_O_2_

Most biological molecules that do not bind transition metal ions do not react directly with H_2_O_2_. However, thiol and cysteine residues of proteins, as well as low-molecular-weight thiols, can directly react with H_2_O_2_ [67]. The reaction of H_2_O_2_ with thiols (RS) depends strongly on the pK_a_ value of the thiol, because the reaction exclusively proceeds via the thiolate anion to form sulfenic acid (RSOH), Reaction (53).

RS^−^ + H^+^ + H_2_O_2_ → RSOH + H_2_O (53)

The rate constants of Reaction (53) range from 0.16 to 10^7^ M^−1^ s^−1^. Sulfenic acids have a lower pKa than the corresponding thiols [79]. Sulfenic acid can react with another thiol or H_2_O_2_ to give the corresponding disulfide (RSSR) or sulfinic acid (RSO_2_^−^), Reactions (54) and (55), respectively. However, the second-order rate constant of Reaction (55) is about 10^3^ times lower than that of Reaction (54) [74,80].

RSOH + RSH → RSSR +H_2_O (54)

RSO^−^ + H^+^ + H_2_O_2_ → RSO_2_^−^ + H_2_O (55)

The second-order rate constants of Reaction (53) for free GSH, cysteine and thioredoxin (TRX) are 0.89 M^−1^ s^−1^, 2.9 M^−1^ s^−1^ and 1.05 M^−1^ s^−1^, respectively [74,81]. However, H_2_O_2_ can react efficiently with peroxiredoxins (PRX); the second order rate constant is 10^7^–10^8^ M^−1^ s^−1^ [74]. H_2_O_2_ reacts with pyruvate to form acetate and CO_2_ with a rate constant of 2.2 M^−1^ s^−1^. The reaction of H_2_O_2_ proceeds via a two-electron pathway: (i) reversible formation of a tetrahedral intermediate; (ii) irreversible decomposition of the intermediate to CO_2_, acetate, and water, Reaction (56) [82].

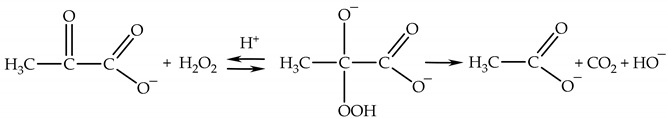
(56)

H_2_O_2_ reacts with carbon dioxide to form peroxymonocarbonate (HCO_4_^−^) with a second-order rate constant for the forward reaction of 2 × 10^−2^ M^−1^ s^−1^, Reaction (57) [74].

H_2_O_2_ + CO_2_ ∆ HCO_4_^−^ + H^+^ (57)

Carbonic anhydrase significantly accelerates Reaction (57) [83]. Transition metals (M) like iron and copper react with H_2_O_2_ via the Fenton mechanism, in which the transition metal cleaves the O–O bond to form HO^•^ and HO^−^, Reaction (58).

M^+(n−1)^ + H_2_O_2_ → M^+n^ + OH^−^ + HO^•^ (58)

Rate constants of the Fenton reaction depend on the metal or metal complex and are in the 5–20 × 10^3^ M^−1^ s^−1^ range [84,85,86,87]. In addition to Reaction (58), the interaction of H_2_O_2_ with transition metals or metal complexes leads to the formation of a higher oxidation state of the metal as L-M(H_2_O_2_)^n+^, L-M^(n+2)+^ or L-MO^(n+2)+^ Reactions (59)–(61) respectively, where L is a ligand of the metal [74,88,89].

L-M^n+^ + H_2_O_2_ → L-M-(H_2_O_2_)^n+^ (59)

L-M-(H_2_O_2_)^n+^ → L-M^(n+2)+^ + 2HO^−^ (60)

L-M-(H_2_O_2_)^n+^ → L-MO^(n+2)+^ + H_2_O (61)

H_2_O_2_ reacts rapidly with heme peroxidases, for example, myeloperoxidase and lactoperoxidase, with a second order rate constant in the range of 10^7^–10^8^ M^−1^ s^−1^ [90]. The rate constant of the reaction of H_2_O_2_ with ascorbate peroxidase (APX) was estimated to be 10^7^ M^−1^ s^−1^ with K_m_ for H_2_O_2_ of 80 µM [91,92].

The scavenging of H_2_O_2_ by peroxidases (PX) proceeds via the peroxidase mechanism (Reactions (62)–(64)) [93].

PX-Fe(III)-porphyrin + H_2_O_2_ → PX-Fe(IV)=O-porphyrin^+^ + H_2_O (62)

PX-Fe(IV)=O- porphyrin^+^ + AH → PX-Fe(IV)=O- porphyrin + A^•^ +H^+^ (63)

PX-Fe(IV)=O- porphyrin + AH → PX-Fe(III)- porphyrin + A^•^ + OH^−^, (64)

where A is a reductant, e.g., AscH_2_.

Catalase (CAT)-dependent scavenging of H_2_O_2_ occurs via a ping-pong mechanism, Reaction (65) and (66), where one H_2_O_2_ molecule is used as an electron donor.

H_2_O_2_ + Fe(III)-CAT → H_2_O + Fe(IV)=O-CAT^+^ (65)

H_2_O_2_ + Fe(IV)=O-CAT^+^→ H_2_O + Fe(III)-CAT + O_2_ (66)

#### 2.3.3. Lifetime and Diffusion Distance of H_2_O_2_

H_2_O_2_ is a small and neutral molecule that can readily diffuse from the site of its production. However, the diffusion of H_2_O_2_ through membranes is hindered [94,95,96] and aquaporins may regulate the diffusion of H_2_O_2_ across membranes. H_2_O_2_ is rather stable and its lifetime in cells is limited by scavenging enzymes and other substances reacting with H_2_O_2_. The lifetime of H_2_O_2_ is 1–3 min in mammalian cells [97] and around 10 s in *Arabidopsis* guard cells [98].

### 2.4. Hydroxyl Radical, HO^•^

HO^•^ has one unpaired electron and is one of the most powerful oxidizing agents. HO^•^ is able to react unselectively with surrounding organic molecules due to the very high positive redox potential, of the pair HO^•^/H_2_O, *E*_0_′ of 2.3 V [99]. The rate constants of reactions of HO^•^ with many molecules are estimated to be larger than 10^9^ M^−1^ s^−1^ [100,101,102].

#### 2.4.1. Formation of HO^•^

The best-known reaction producing HO^•^ is the Fenton reaction, in which the O–O bond of H_2_O_2_ is cleaved by reaction with a transition metal ion (Reaction (58)). HO^•^ is produced via the same mechanism in the reaction of H_2_O_2_ with O_2_^•−^ (metal-catalyzed Haber–Weiss reaction), Reaction (3).

Another well-known means of HO^•^ generation is through the photolysis of oxygen-containing species. In aqueous solution, the nitrate anion (NO_3_^−^) can absorb UV radiation and produce HO^•^, Reactions ((67) and (69). The formation of HO^•^ is also observed upon photolysis of the nitrite ion (NO_2_^−^), Reactions (68) and (69) [102,103]. Due to the requirement of short-wavelength UV radiation, this process does not occur in biological systems.

NO_3_^−^ + hν ⇆ + [O^•−^–^•^NO_2_] → O^•−^ + ^•^NO_2_ (67)

NO_2_^−^ + hν → O^•−^ + ^•^NO_2_ (68)

O^•−^ + H^+^ ⇆ HO^•^ (69)

The photolysis of a H_2_O_2_ molecule gives two HO^•^ with a quantum yield of approximately 0.5 in aqueous solutions, Reaction (70). H_2_O_2_ photolysis requires UV-C radiation because the molar absorption coefficient of H_2_O_2_ is very low above 300 nm. H_2_O_2_ photolysis is an effective way of generating HO^•^ in aqueous solutions [102,104].

H_2_O_2_ ∆ [HO^•^–HO^•^] → 2HO^•^ (70)

Another potential source of HO^•^ is O_3_. The addition of an electron to an O_3_ molecule leads to the decomposition of O_3_ to HO^•^ and O_2_ via the formation of an ozonide anion radical [105]. O_3_ can also be decomposed to O_2_ and HO^•^ via reduction by exited chlorophyll (Chl*), Reaction (71) [102].

O_3_ + Chl* + H^+^ → O_2_ + HO^•^ + Chl^+^ (71)

However, O_3_ has not been found inside plant cells.

O_3_ reacts with O_2_^•−^ to form HO^•^ and O_2_ as final products, Reaction (72)–(74)

O_3_ + O_2_^•−^ → O_3_^•−^ + O_2_ (72)

O_3_^•−^ + H^+^ → HO_3_^•^ (73)

HO_3_^•^ → HO^•^ + O_2_ (74)

HO^•^ can be also produced in a radical–radical reaction of HO_2_^•^ with RO_2_^•^, Reaction (75) [102].

RO_2_^•^ + HO_2_^•^ → RO^•^ + HO^•^ + O_2_ (75)

#### 2.4.2. Reactions of HO^•^

HO^•^ participates in several typical reactions:

*abstraction of hydrogen atom* from an organic molecule (RH) with the formation of H_2_O and radical (R^•^) of substrate (76);

HO^•^ + RH → H_2_O + R^•^ (76)

*addition to double bonds* with the formation of a hydroxylated radical (77);

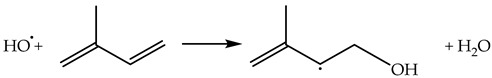
(77)

*electron transfer reactions* leading to the formation of a neutral radical (78) or a cation radical (79) [106]; SCN^−^ is the thiocyanate ion.

HO^•^ + SCN^−^ + ^−^OH + SCN (78)

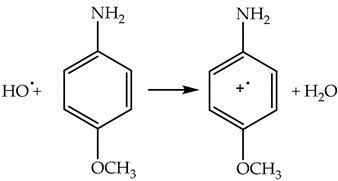
(79)

*Formation of an aromatic-OH adduct* due to a reaction of an aromatic compound with HO^•^ is one of the methods for HO^•^ detection with high-performance liquid chromatography–mass spectrometry. For example, HO^•^ can react with phenylalanine to form isomers of tyrosine, Reaction (80) [107]. Isomers of tyrosine are rather stable and not normally present in proteins, and can serve as HO^•^ traps in biological samples [108].

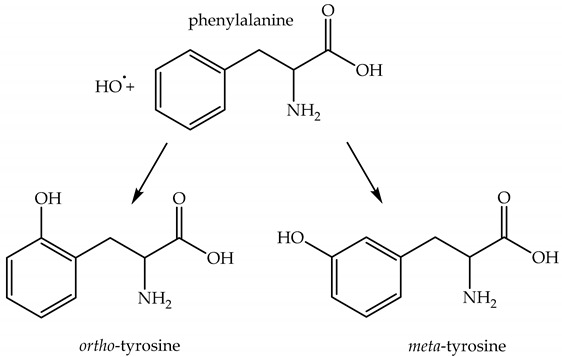
(80)

HO^•^ interacts with many metal (M) cations via an electron transfer Reaction (81), with a rate constant of ~10^8^ M^−1^ s^−1^ [100].

HO^•^ + M^n+^ → M^(n+1)+^ + OH^−^ (81)

HO^•^ initiates lipid peroxidation, resulting in hydrogen abstraction from a pentyl group of an unsaturated fatty acid, and the formation of a radical that interacts with O_2_ to form an ROO^•^ with a rate constant of ~10^8^ M^−1^ s^−1^ [109], Reaction (82).

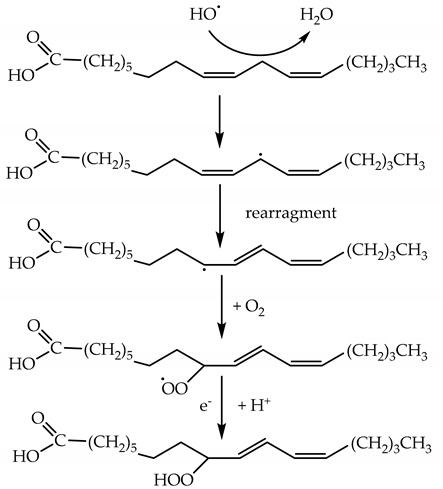
(82)

#### 2.4.3. Lifetime and Diffusion Distance of HO^•^

The lifetime of HO^•^ in aqueous solution has been estimated to range from picoseconds to nanoseconds. The self-diffusion coefficient of HO^•^ in water has been estimated to be 2.8 × 10^−5^ cm^2^ s^−1^, and consequently the diffusion distance of HO^•^ is a few molecular diameters from the site of origin [110,111].

## 3. Production of ROS in Chloroplasts

Chloroplasts have a high metabolic activity accompanied with intensive formation of redox active compounds, which are able to react with oxygen to produce ROS. Most ROS production in the chloroplast occurs by the components of the light reactions. Photorespiration is responsible for 70% of total H_2_O_2_ production in the leaves of C3 plants [112,113], but this reaction runs in peroxisomes outside of the chloroplast.

### 3.1. ROS Production in Chloroplast Stroma

#### 3.1.1. Formation of ^1^O_2_ in the Stroma

The chloroplast stroma is not considered as a significant source of ^1^O_2_, although disintegration of the antenna complexes under stress conditions and disturbances in Chl synthesis and the accumulation of its precursors may lead to ^1^O_2_ production in the stroma [114]. The lack of FLU, a nuclear-encoded chloroplast protein that plays a key role during the negative feedback control of Chl biosynthesis, leads to the accumulation of protochlorophyllide in plastids and, consequently, to photosensitized generation of ^1^O_2_ [115]. It has been recently shown that lipoxygenase localized in the chloroplast is responsible for ^1^O_2_ formation [116]. Lipoxygenase initiates lipid oxidation to corresponding lipid peroxides, which decompose to lipid peroxyl radicals (LOO^•^). LOO^•^ can react with each other, forming a cyclic endoperoxide (dioxetane) intermediate. Dioxetane, in turn, can decompose via the Russel mechanism to form ^1^O_2_, Reaction (9).

Theoretically, the Haber-Weiss mechanism (Reactions (3) and (4)) can cause the formation of ^1^O_2_ in the stroma, but the rate of this reaction is expected to be low due to very efficient scavenging of O_2_^•−^ by chloroplasts [12] and the low rate constant of Reactions (3) and (4) [21].

#### 3.1.2. Formation of Reduced Forms of Oxygen, O_2_^•−^, H_2_O_2_, HO^•^, by Fd in the Stroma

Ferredoxin (Fd) and free flavins (FL) and flavoenzymes are considered as the main stromal components involved in O_2_ reduction and ROS formation. Fd is involved in electron transfer from the acceptor side of PSI to NADP^+^ in a reaction catalyzed by Fd-NADP^+^ reductase (FNR) (EC 1.18.1.2) [117]. Fd is a soluble 10 kDa protein [118] containing a 2Fe-2S center [119]. The leaf-type Fd from higher plants has an *E_m_* vs. NHE (at pH 8.0) from −390 to −425 mV [120]. The redox potential of Fd_ox_/Fd_red_ is much more negative than the redox potential of O_2_/O_2_^•−^ (−160–−180 mV) in aqueous solutions, and the reduction in O_2_ by Fd is thermodynamically very favorable. It was suggested that the oxidation of Fd can occur via 2-step reaction with the formation of the Fd-O_2_^•−^ complex [121], Reactions (83) and (84).

Fd(II) + O_2_ ∆ Fd(II)-O_2_ (83)

Fd(II)-O_2_ ∆ Fd(III)-O_2_^•−^ (84)

The subsequent dissociation of the Fd(III)-O_2_^•−^ yields O_2_^•−^. The involvement of Fd in O_2_ reduction has been studied by the addition of Fd to a suspension of thylakoid membranes in the light. In this case, Fd is reduced by PSI. Fd increased the rate of O_2_ consumption in response to an increase in Fd concentration. The rate of O_2_ consumption was saturated at 60 μM Fd and became sevenfold higher than in the absence of Fd [122,123]. On the other hand, Fd did not stimulate the formation of O_2_^•−^ measured using O_2_^•−^ traps such as epinephrine or cytochrome *c* [124]. This suggests that the formation of O_2_^•−^ by exogenous Fd is very slow in comparison to the formation of O_2_^•−^ by thylakoid-bound photoreductants. This correlates with the finding that the rate of direct oxidation of Fd by O_2_ was rather low, with a second-order rate constant of 10^3^ M^−1^ s^−1^ for chemically (dithionite as reductant) reduced Fd [125]. Recent studies show that O_2_ reduction in a thylakoid suspension in the presence of Fd is a result of O_2_ reduction by both a membrane-bound reductant and Fd. The distribution of electron flow from Fd and membrane-bound reductant to O_2_ is sensitive to light intensity and NADP^+^ but not to Fd concentration. Furthermore, Fd stimulates the reduction in O_2_ by membrane-bound reductants [126]. Interestingly, NADP^+^ very strongly inhibits O_2_ reduction by Fd but stimulates O_2_ reduction by a thylakoid-membrane-bound reductant [126]. These results suggest that Fd has a minor role in the direct reduction of O_2_ in vivo.

Catalase, added to a suspension of illuminated thylakoid membranes, almost completely suppressed Fd-dependent O_2_ consumption, suggesting that H_2_O_2_ is the final product of O_2_ reduction by Fd. This is clear from the well-known stoichiometry between O_2_ consumption and O_2_ evolution in isolated thylakoids when a reduction in O_2_ occurs by electrons originally arising from water-splitting in PSII without any electron acceptors or ROS traps, Reactions (85)–(89). In this case, H_2_O_2_ is produced via dismutation of O_2_^•−^ [127,128].

2H_2_O → O_2_ + 4H^+^ + 4e^−^  water splitting in PSII (85)

4Fd(III) + 4e^−^ → 4Fd(II)  Fd reduction in PSI, (86)

4Fd(II) + O_2_ → 4Fd(III) + 4O_2_^•−^  O_2_^•−^ formation, (87)

4O_2_^•−^+ 4H^+^ → 2H_2_O_2_ + 2O_2_   O_2_^•−^ dismutation, (88)

2H_2_O_2_ → 2H_2_O + O_2_  H_2_O_2_ decomposition, (89)

Reaction (84) is included only to describe an experiment with isolated thylakoids. Chloroplasts do not contain CAT and, in chloroplasts, H_2_O_2_ is scavenged by APX (Reactions (62)–(64)) [12]. Fd-mediated photosynthetic O_2_ consumption is inhibited by SOD, suggesting that autoxidation of Fd is involved in both the reduction of O_2_ to O_2_^•−^ and of O_2_^•−^ to H_2_O_2_, Reactions (90) and (91) [129].

Fd(II) +O_2_ → Fd(III) + O_2_^•−^ (90)

Fd(II) + O_2_^•−^ + 2H^+^ → Fd(III) + H_2_O_2_ (91)

The reduction in O_2_^•−^ by Fd can produce H_2_O_2_ in the chloroplast stroma. The very low stimulation of O_2_^•−^ production by Fd in comparison to O_2_ consumption [124,127] could result from Reaction (91). It is also possible that Fd stimulates O_2_ reduction in chloroplasts through a more complicated process than the direct reduction in O_2_.

#### 3.1.3. Formation of Reduced Forms of Oxygen, O_2_^•−^, H_2_O_2_, HO^•^, by Flavins in the Stroma

The reduction in O_2_ by FLs can also produce ROS in the stroma, as FLs react with O_2_ to form O_2_^•−^ and H_2_O_2_. Free FLs are oxidized by O_2_ via the formation of an intermediate complex containing a radical pair that can decompose with the formation of a flavin semiquinone radical (FLH^•^) and O_2_^•−^, Reaction (92). The radical pair may also be transformed to a flavin hydroperoxide (FLHOOH) that can decompose to a FL and H_2_O_2_, Reaction (93) [130,131,132,133,134].

FLH^−^ + O_2_ → [FLH^•^-OO^•−^] → FLH^•^ + O_2_^•−^ (92)

[FLH^•^-OO^•−^] + H^+^ → FLHOOH → FL + H_2_O_2_ (93)

The second-order rate constant of Reaction (92) is estimated to be only 2.5 × 10^2^ M^−1^ s^−1^ [133,134]. The formation of FLH^•^ (Reaction (92)) can also initiate complex autocatalytic FL oxidation. In solution, some amount of the FLH^•^ is formed in a mixture of oxidized and reduced FL (FLH_2_) via an equilibrium reaction, Reaction (94).

FLH_2_ + FL ∆ 2FLH^•^ (94)

With free FLs, the second-order rate constants for forward and backward Reaction (94) are 10^6^ M^−1^ s^−1^ and 5 × 10^8^ M^−1^ s^−1^, respectively [133]. Thus, the equilibrium constant of Reaction (94) is 2 × 10^−3^. Only 1–5% of the radical is stabilized in an equimolar mixture of oxidized and reduced FL [134,135]. The flavin semiquinone radical can exist in neutral (FLH^•^) or anionic form (FL^•−^), with a pKa of ≈8.5 (Reaction (95)).

FLH^•^ ∆ FL^•−^ + H^+^ (95)

The next steps of the autocatalytic process are described by Reactions (96)–(101), in which the oxidation of FLH^•^ or FL^•−^ by O_2_ produces O_2_^•−^, Reaction (96) and (97), and then O_2_^•−^ or HO_2_^•^ can react with FLH_2_ to form FLH^•^ or FL^•−^ and H_2_O_2_, Reaction (98) and (99). O_2_^•−^ and HO_2_^•^ can also react with FLH^•^ to form FL and HO_2_^−^ or H_2_O_2_, Reaction (100) and (101).

FLH^•^ + O_2_ → FL + O_2_^•−^ + H^+^ → FL + HO_2_ (96)

FL^•−^ + O_2_ → FL + O_2_^•−^ (97)

FLH_2_ + O_2_^•−^ → FL^•−^ + H_2_O_2_ (98)

FLH_2_ + HO_2_^•^ → FLH^•^ + H_2_O_2_ (99)

FLH^•^ + O_2_^•−^ → FL + HO_2_^−^ (100)

FLH^•^ + HO_2_^•^ → FL + H_2_O_2_ (101)

The second-order rate constant for the reaction of O_2_ with FLH^•^ (Reaction (96)) is around 10^4^ M^−1^ s^−1^ and that of the reaction of O_2_ with FL^•−^ (Reaction (97)) is much larger, around 10^8^ M^−1^ s^−1^ [133]. The *E_m_* at pH 7 for FL/FLH^•^ of FL mononucleotide is estimated to be −313 mV vs. NHE, which is more negative than the redox potential of O_2_/O_2_^•−^ (−160 mV) in aqueous solution [134,136]. As predicted from the redox potentials, the reaction of FL^•−^ with O_2_ is thermodynamically favorable and the rate of oxidation of FLs by O_2_ via autocatalytic mechanisms can strongly depend on both the stability and pKa value of FLH^•^. Free flavins can therefore be involved in the formation of ROS in the chloroplast stroma.

Flavoenzymes can also be involved in the production of O_2_^•−^ and H_2_O_2_ in chloroplasts. The reactivity of FLs in flavoenzymes is modulated by the protein environment of reduced FLs and the second-order rate constant of O_2_ reduction by flavoenzymes varies from 2 M^−1^ s^−1^ to 2 × 10^6^ M^−1^ s^−1^ [134]. The redox potential of flavoenzymes vs. NHE varies from −16 to −263 mV and −60 to −231 mV for FL/FLH^•^ and for FLH^•^/FLH_2_, respectively [134]. The reactivity of flavoenzymes towards O_2_ may differ by several orders of magnitude between flavoenzymes having similar redox potentials [134]. Such very high differences are due to the protein environment, which affects both O_2_ movement and the binding of O_2_ to the active site. In addition, the polarity of the protein environment in the active site can change the redox potential of O_2_/O_2_^•−^ because the redox potential of O_2_/O_2_^•−^ becomes very negative (≈−600 mV vs. NHE) in a non-polar solvent [55]. The reduction in O_2_ by a FL in a non-polar active site is thus unlikely [134]. The reactivity of flavoenzymes towards O_2_ depends on the stabilization of the semiquinone in the active site because semiquinones show higher reactivity with O_2_ than fully reduced FLs [133]. The reactivities of flavoenzymes with O_2_ can be limited by a substrate that acts as a specific electron acceptor of the flavoenzyme. Some flavoenzymes, like glucose oxidase and xanthine oxidase, employ O_2_ as a natural acceptor, forming both H_2_O_2_ and O_2_^•−^ with high efficiency [137,138]. The mechanism of O_2_ reduction by flavoenzymes has recently been reviewed in detail [139].

Some stromal flavoenzymes, such as FNR, monodehydroascorbate reductase (MDAR), glutathione reductase (GR) and glycolate oxidase, can efficiency reduce O_2_ to O_2_^•−^ in the absence of the specific substrate [12,140]. The flavoenzymes are reduced and oxidized by their specific electron donors and acceptors with high rates. For example, MDAR is reduced by NAD(P)H with a second-order rate constant of 1.8 × 10^8^ M^−1^ s^−1^ [141], and the reduced MDAR can be oxidized by monodehydroascorbate radical (AscH^•^ and its anionic form (Asc^•−^), abbreviated as MDA) with a second-order rate constant of 2.6 × 10^8^ M^−1^ s^−1^ [142]. The flavoenzymes MDAR, GR, FNR and glycolate oxidase can, after reduction by Fd or NAD(P)H, efficiently reduce O_2_ to form O_2_^•−^ as a primary product with a maximum rate in isolated thylakoids of 300 μmol of O_2_^•−^ (mg Chl)^−1^ h^−1^. The K_m_ value for O_2_ in the reaction with MDAR was 100 μM, while the K_m_ for O_2_ reduction by thylakoid membranes was 10 μM [140]. In the work of Goetze and Carpentier [143] the addition of FNR to a thylakoid suspension increased H_2_O_2_ formation by 33%. The rates of O_2_ consumption in the absence and presence of FNR were 28 and 37 μmol (mg Chl)^−1^ h^−1^, respectively, which imply (in a system that produces O_2_ at PSII) O_2_^•−^ production rates of 112 μmol (mg Chl)^−1^ h^−1^ and 148 μmol (mg Chl)^−1^ h^−1^, respectively [143]. However, the autoxidation rates of the MDAR, glutathione reductase and FNR that can be reduced by NAD(P)H are extremely slow [141,144], suggesting that the high rates of O_2_ reduction by photoreduced flavoenzymes result from the formation of a stable semiquinone. Photoreduction of the flavoenzymes, added to thylakoid membranes, can occur at the F_A_/F_B_ (4Fe-4S clusters of PSI) [140]. The prosthetic group of MDAR can be reduced by F_A_/F_B_ to the semiquinone form, as the *E_m_* of F_A_/F_A_^−^ and F_B_/F_B_^−^ pairs are −479 and −539 mV, respectively [145]. The stable semiquinones of flavoenzymes can be oxidized by O_2_ with a high rate [133]. In leaves, the photoreduction rate of O_2_ was estimated to be 18–26 μmol O_2_ (m^−2^ of leaf area) s^−1^ [146] which gives a rate of O_2_^•−^ production of 240–350 μmol (mg Chl)^−1^ h^−1^ assuming 0.6 mmol Chl (m leaf area)^−2^ [147]. Thus, flavoenzymes may contribute to the high rates of O_2_ photoreduction in chloroplasts.

In cyanobacteria [148,149], flavodiiron proteins reduce oxygen to water without ROS production. A substantial fraction of the total photosynthetic electron flow may be directed to this route [150]. Flavodiiron proteins have later been found from all oxygenic phototrophs except for angiosperms and some non-green algae (for review, see [151]).

#### 3.1.4. Formation of H_2_O_2_ in the Stroma

In most cases, the reduction in O_2_ by Fd or by flavoenzymes yields O_2_^•−^ as the primary product [127,140] and H_2_O_2_ is usually formed via the dismutation of O_2_^•−^ [152]. The dismutation of O_2_^•−^ in Reaction (5) is a major mechanism of H_2_O_2_ formation in the stroma. At physiological pH, the rate constant for O_2_^•−^ dismutation is about 10^5^ M^−1^ s^−1^ [68]. SOD catalyzes the disproportionation of O_2_^•−^ at a diffusion-controlled rate, as the second-order rate constant was estimated to be 2.2 × 10^8^ M^−1^ s^−1^ for stromal conditions [152]. H_2_O_2_ can also be formed via a reaction of O_2_^•−^ with a reduced stromal compound like AscH_2_, GSH or Fd [127,152]. The yields of these reactions are very small in the presence of SOD. The second-order rate constants of reduction of O_2_^•−^ with AscH_2_ and GSH were estimated to be 3.3 × 10^5^ M^−1^ s^−1^ and 10^3^ M^−1^ s^−1^, respectively [62,66].

In chloroplasts, H_2_O_2_ is efficiently scavenged by an enzyme-catalyzed reduction in H_2_O_2_ by AscH_2_ to form H_2_O and DHA (see Section 5.1). The reaction is catalyzed by both stromal and thylakoid-bound APXs [5,152]. Accumulation of H_2_O_2_ can lead to the generation of HO^•^ via the Fenton reaction (Reaction (58)) if the scavenging of H_2_O_2_ by the antioxidant enzymes is not fast enough for the efficient removal of H_2_O_2_. The Fenton reaction is possible in the chloroplast because up to 80% of cellular Fe in leaf cells is found in chloroplasts [153]. The involvement of free Fe in Fenton reaction is limited, since the Fe is stored in a redox inactive form as ferritin [154,155]. However, Fe can be activated and released from ferritin via interaction of ferritin with O_2_^•−^ [156]. In addition to free transition metals, Fd can be involved in the production of HO^•^ [157,158]. The second-order rate constant for the reaction of reduced Fd with H_2_O_2_ was found to be 5 × 10^3^ M^−1^ s^−1^ [121], which is two orders of magnitude higher than the second-order rate constant of HO^•^ production in the Fenton reaction, 84 M^−1^ s^−1^ [84].

### 3.2. Formation of ROS in Thylakoid Membranes

The PETC employs three membrane protein complexes: PSI, PSII, their respective light-harvesting complexes (LHCI and LHCII), and the cytochrome b6/f complex (Cyt b6f, a plastoquinol-plastocyanin-oxidoreductase). Electron transfer between the complexes involves two mobile electron carriers, PQ and plastocyanin (PC). The liposoluble PQ mediates electron flow from PSII to Cyt b6f complex, and the water-soluble lumenal protein PC mediates electron flow from Cyt b6f to PSI. ROS are formed in several sites of the PETC, including PSII, PSI, the PQ pool and the light-harvesting complexes (LHC).

#### 3.2.1. Formation of ^1^O_2_ in Thylakoids

The production of ^1^O_2_ in plants occurs mainly by the interaction of O_2_ with excited states of Chls (where ^1^Chl* and ^3^Chl are the excited singlet and triplet states of Chl, respectively) via spin-conserved reactions, (Reactions (102) and (103)).

^1^Chl* + O_2_ → ^3^Chl + ^1^O_2_ (102)

^3^Chl + O_2_ → ^1^Chl + ^1^O_2_ (103)

Reaction (102) is negligible because the lifetime of ^1^Chl* is very short (~10^−8^ s) [137]. The lifetime of ^3^Chl is around 10^−3^ s under anaerobic conditions [16,45]. In solution, the quenching of ^3^Chl by O_2_ mostly occurs via ^1^O_2_ generation with a second-order rate constant of 2 × 10^9^ M^−1^ s^−1^ [16]. ^3^Chl is formed both in the LHCs and in the reaction centers (RC) of PSII and PSI. In the LHCs, ^3^Chl is formed by intersystem crossing (ISC) from ^1^Chl* [114,159] and in the RCs by charge recombination. In PSII, the charge recombination between P680^+^ (the primary donor) and Q_A_^−^ (bound quinone) produces ^3^P680 [114,160]. ^3^P680 is formed through a time-dependent “virtual triplet state” of the primary radical pair P680^+^ Pheophytin (Pheo)^−^ [161]. A triplet state of P700, the primary donor of PSI, can also be formed via charge recombination [162].

Chls are mostly bound to LHCII and CP47 and CP43 proteins of PSII and the PSA A/B proteins of PSI. According to the high concentration of Chl in chloroplasts, around 60 mM [163], a significant formation of ^3^Chl via ISC in LHCs should be observed. However, there is no experimental evidence for the production of ^1^O_2_ by the formation of ^3^Chl in LHCs in vivo [114]. The formation of both ^3^Chl and ^1^O_2_ has been observed in isolated LHCs. The formation of ^1^O_2_ was found in isolated LHCII with an electron paramagnetic resonance (EPR) measurement of 2,2,6,6-tetramethylpiperidine as a spin trap of ^1^O_2_ [164,165]. The appearance of long-lived ^3^Chl in LHCs has been suggested to result from a small population of Chls that are substantially uncoupled from the matrix of LHC [166,167]. In a reconstructed Chl-protein complex, light-dependent ^1^O_2_ formation is lower by a factor of four compared to free Chl [168]. As the isolated protein does not contain pigments that would effectively quench ^3^Chl or ^1^O_2_, it was suggested that the low ^1^O_2_ formation is caused by the tight packing of Chl molecules inside the hydrophobic zone of the pigment–protein complex where the interaction of ^3^Chl with O_2_ is limited [168]. The same situation can probably be realized in LHCs where Chls are tightly packed. Furthermore, LHCs contain carotenoids that efficiently quench ^3^Chl. In addition, the highly efficient transfer of excitation energy to the RC lowers the probability of ISC. Thus, ^1^O_2_ can only be formed in LHCs in sites where Chl is weakly bound to the protein matrix and ^3^Chl cannot be efficiently quenched by carotenoids.

The main source of ^1^O_2_ appears to be O_2_ reacting with ^3^P680. As in the case of antenna complexes, the formation of ^1^O_2_ in the RC also depends on two factors: the lifetime of ^3^P680 and the probability that O_2_ reacts with ^3^P680. Assuming that the accessibility of O_2_ to ^3^P680 is not changed significantly in different conditions; the yield of ^1^O_2_ generation in the RC of PSII is mainly limited by the rate of ^3^P680 formation. The formation of ^3^P680 proceeds via charge separation and charge recombination. The formation of the excited singlet state ^1^P680* is followed by the formation of the radical pair [P680^+^Pheo^−^] [169]. In the next step, an electron from Pheo^−^ is transferred to Q_A_ and the pair [P680^+^PheoQ_A_^−^] is formed. P680^+^Pheo^−^ can recombine to yield ^3^P680, and the formation of ^1^O_2_ with a high yield was observed in an isolated PSII RC lacking Q_A_ and a functional donor side [114,170,171]. In thylakoid membranes where both the donor and acceptor side are functional, electron transfer to Q_A_ and then to Q_B_ prevents the formation of a long-lived primary radical pair [172]. However, pairs [P680^+^Pheo^−^] and [P680^+^PheoQ_A_^−^] recombine when forward electron transfer is impossible. A fraction of the recombination of the pair [P680^+^PheoQ_A_^−^] produces [P680^+^Pheo^−^Q_A_] [173]. Originally, [P680^+^Pheo^−^] is formed from [^1^P680*Pheo] in a virtual singlet state ^1^[P680^+^Pheo^−^] that recombines to ^1^P680*. However, the long lifetime of the state [P680^+^PheoQ_A_^−^] destroys spin correlation, and therefore the recombination [P680^+^PheoQ_A_^−^] to [P680^+^Pheo^−^Q_A_] often produces a virtual triplet state of the primary radical pair ^3^[P680^+^Pheo^−^Q_A_] that has such a spin configuration that its recombination to an excited state of the primary donor produces a triplet, ^3^P680 [174]. At 40 K, the triplet state is mainly localized on the monomeric chlorophyll Chl_D1_ [175], while, at 25 °C, about 30% of this state is associated with the chlorophyll P_D1_ [176]. Thus, the site of ^1^O_2_ production is mainly localized in the D1 protein of PSII. The formation of ^3^P680 has a high probability because the two β-carotene molecules of the PSII RC are located far from Chl_D1_ and P_D1_, at 19.9 Å and around 30 Å, respectively [174,177]. Such a long distance does not allow for the direct quenching of ^3^P680 [178].

^1^O_2_ generation by photosynthetic samples has been measured using several methods, including the spin traps 2,2,6,6-tetramethylpiperidine [179,180,181,182,183,184,185], 2,2,6,6-tetramethyl-4-piperidone [183,184,186], 3-[*N*-(β-diethylaminoethyl)-*N*-dansyl]aminomethyl-2,2,5,5-tetramethyl-2,5-dihydro-1H-pyrrole [187,188,189], trans-1-(2′-methoxyvinyl)pyrene [190], histidine [191,192,193] and Singlet Oxygen Sensor Green [190,194,195]. The 1270 nm luminescence has been used to measure ^1^O_2_ generation by isolated RC complexes [171,196]. The methods of ^1^O_2_ measurement were recently reviewed [23].

The absolute rate of ^1^O_2_ production can be estimated by comparing the signal strength (e.g., 1270 nm luminescence intensity, fluorescence yield, the yield of 2,2,6,6-tetramethylpiperidine-1yl) oxyl or EPR signal amplitude) with the signal obtained from a sensitizer chemical with a known ^1^O_2_ yield. The main limitation of such an estimation is that, in photosynthetic material, ^1^O_2_ may be effectively quenched before reacting with the sensor, and therefore all estimates of ^1^O_2_ yield of photosynthetic material represent a lower limit. At the PPFD (photosynthetic photon flux density) of 2000 µmol m^−2^s^−1^, histidine-dependent O_2_ uptake measurements showed that isolated PSII RCs (6 Chl/RC, [197]) produce ^1^O_2_ at a rate of 4000 µmol ^1^O_2_ (mg Chl)^−1^ h^−1^, with a quantum yield 0.16 [191]. The yield of ^1^O_2_ per ^3^P680 is very high, as the quantum yield of ^3^P680 formation in the same preparations was 0.3 [191,198]. 2,2,6,6-tetramethylpiperidine measurements at the same PPFD showed that isolated thylakoid membranes produced 3.73 × 10^−7 1^O_2_ molecules per Chl molecule s^−1^, and the quantum yield of ^1^O_2_ formation was 2.59 × 10^−4^ [185]. Assuming 490 and 173 Chls per PSII and PSI, respectively [197], and a PSII:PSI ratio of 1 [199], the ratio of Chl to RC of PSII is 663 for a plant thylakoid membrane. Thus, isolated RCs and thylakoids produce, at PPFD 2000 µmol m^−2^s^−1^, 21,600 and 0.89 ^1^O_2_ per RC per h, respectively. The large difference probably reflects differences in both the actual ^1^O_2_ production rate and in the experimental method. In cyanobacteria illuminated at PPFD 2300 µmol m^−2^ s^−1^ in deuterium oxide, a decrease in O_2_ concentration in the presence of histidine showed ^1^O_2_ production of approximately 27 µmol (mg Chl)^−1^ h^−1^ [192], suggesting that ^1^O_2_ production in vivo may actually be of the same order of magnitude as the maximum rate of O_2_ evolution. A similar conclusion was drawn from measurements with isolated RCs [191].

Inactivation of the oxygen-evolving complex (OEC) of PSII leads to an increase in the redox potential of the Q_A_/Q_A_^−^ pair, so that ^3^P680 is no longer formed, and therefore virtually no ^1^O_2_ can be produced through recombination reactions. However, even in this situation, some ^1^O_2_ production can be expected because inactivation of the Mn-cluster leads to the oxidation of organic molecules, presumably by P680^+^ or TyrZ^•+^ (the redox active tyrosine residue in the D1 protein), and the formation of organic hydroperoxides [200]. Recently, ^1^O_2_ formation has been detected in Mn-depleted PSII membranes and correlated with R^•^ formation on the donor side of PSII. It was proposed that ^1^O_2_ is formed via the Russell mechanism, Reactions (9), (104) and (105) [201,202].

P680^•+^ (or TyrZ^•^) + RH → P680 (TyrZ) + R^•^ (104)

R^•^ + O_2_ → ROO^•^ (105)

**Figure 1:**
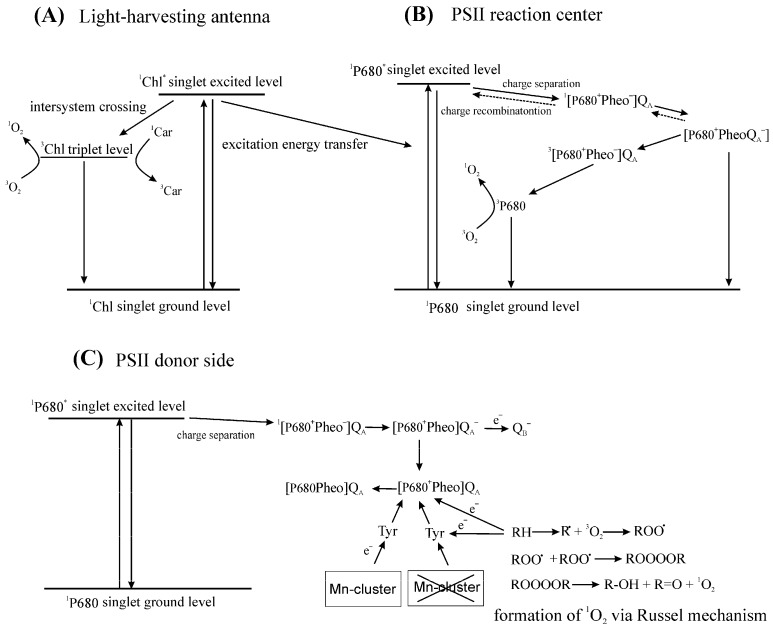
Singlet oxygen (^1^O_2_) generation in the antenna complex (**A**) and in the reaction center of PSII via charge recombination reactions (**B**). Proposed mechanism for the formation of ^1^O_2_ on the donor side of PSII via formation of organic peroxyl radicals (**C**). (**A**) In the antenna complex, the absorption of a photon by a molecule of Chl leads to the formation of a singlet excited state, ^1^Chl*, that can transform to the triplet state ^3^Chl via intersystem crossing. The formation of ^1^O_2_ via intersystem crossing has been demonstrated to occur in isolated LHCII [164,165]. (**B**) In the reaction centre of PSII, [P680^+^Pheo^−^] is originally formed via electron transfer from ^1^P680* to Pheo in a virtual singlet state ^1^[P680^+^Pheo^−^] that recombines to ^1^P680*. Charge recombination of P680^+^PheoQ_A_^−^] causes the formation of ^1^[P680^+^Pheo^−^]. The long lifetime of the state [P680^+^PheoQ_A_^−^] destroys spin correlation, and therefore the recombination [P680^+^PheoQ_A_^−^] to ^1^[P680^+^Pheo^−^Q_A_] often produces a “virtual triplet state” of the primary radical pair, i.e., a radical pair with such a spin configuration that, in its recombination to an excited state of the primary donor, produces a triplet, ^3^P680 [172,173,174]. (**C**) On the donor side of PSII, carbon centered radicals (R^•^) can be formed via the oxidation of lipids and proteins by P680^+^ if electron donation from Mn-cluster to P680^+^ does not function. R^•^ are able to react with O_2_ to form peroxyl radical (ROO^•^). Two peroxyl radicals react with each other to form linear tetraoxide (ROOOOR) that decomposes to ^1^O_2_, carbonyl (R=O) and alcohols (R–OH) via the Russell mechanism [200,201,202].

Thus, the formation of ^1^O_2_ associated with PETC can proceed both via the interaction of O_2_ with ^3^Chl and decomposition of ROOOOR (Reaction (9)). The formation of ^1^O_2_ can occur in at least three sites of PETC: (1) LHCs; (2) RC of PSII; and (3) the donor side of the PSII (Figure 1).

PSI is not considered as a site of ^1^O_2_ production, although theoretically the formation of ^3^Chl can occur through charge recombination between P700^+^ and its electron acceptors. In isolated PSI membrane fragments, the recombination of [P700^+^A_0_^−^] in the presence of dithionite was found to lead to the formation of the triplet state in PSI RCs with a quantum yield of approximately 30% [203]. In PSI particles, the flash-induced absorption changes at 820 nm are attributed to the formation of ^3^P700 via conversion of the cation–anion biradical pair [P700^+^A_0_^−^], with a yield approaching approximately 50% for 10 ns [204]. It was found that an increase in absorption at 820 nm is immediately followed by a multiphasic decay, including a major fast phase within 5–10 µs and an intermediate phase (about 10–15% of the signal) within 2 ms [204]. Interestingly, O_2_ does not affect the decay. It can be speculated that this indicates that O_2_ is unable to efficiently quench ^3^P700 in isolated PSI complexes and thereby produce ^1^O_2_. It seems that, even if ^3^P700 is formed in PSI, its quenching by O_2_ is minimized. It has also been suggested that charge recombination mainly occurs between P700^+^ and phylloquinone A_1_^−^, which minimizes the formation of ^3^P700 triplet [205]. ^1^O_2_ could be detected in PSII membrane fragments and PSII core complexes but not in PSI particles under the same conditions [181]. However, isolated PSI-LHCI supercomplexes of *Arabidopsis* produced ^1^O_2_ at a rate of approximately one tenth of that measured in PSII-LHCII supercomplexes, and the rate of ^1^O_2_ production by PSI-LHCI supercomplexes of the low-carotene *szl1* mutant was approximately one fourth of that measured in PSII-LHCII supercomplexes [206]. There is also some evidence that the Fe-S centers of PSI produce ^1^O_2_ [207].

#### 3.2.2. Oxygen Reduction in PETC

The first evidence that O_2_ can accept electrons from PETC was observed by Mehler who found that O_2_ was consumed and H_2_O_2_ evolved under the illumination of broken chloroplasts [208]. Light-dependent O_2_ consumption as an indicator of Mehler’s reaction has been reported also in vivo in algae and cyanobacteria [209,210], and in isolated intact chloroplasts with the capacity for CO_2_ fixation [211,212]. Later studies have shown that O_2_ reduction occurs in different sites of the PETC (including both PSII and PSI), and illumination of thylakoids triggers the appearance of several forms of reduced O_2,_ including O_2_^•−^, HO_2_^•^, H_2_O_2_ and HO^•^ [213]. Because the acceptor side of PSI is the major site of O_2_ reduction in thylakoid membranes (see reviews [213,214,215]), the term “Mehler’s reaction” has become synonymous with O_2_ reduction at the acceptor side of PSI, with H_2_O_2_ as the final product.

#### 3.2.3. Formation of Reduced Forms of Oxygen, O_2_^•−^, H_2_O_2_, HO^•^, in PSII

Although ^1^O_2_ is the main ROS produced in PSII, O_2_^•−^, H_2_O_2_ and HO^•^ have also been found to be formed [160,174]. The contribution of PSII to the generation of O_2_^•−^ in the intact chloroplast is generally small [216]. The O_2_ consumption associated with O_2_ reduction by PSII membranes capable of water-splitting is about 1 µmol O_2_ (mg Chl)^−1^ h^−1^ or 4 µmol O_2_^•−^ (mg Chl)^−1^ h^−1^ when O_2_ is the only electron acceptor [128]. The rate of O_2_ reduction is higher in disintegrated PSII complexes, which might suggest that, during stress conditions in vivo, when the structure and functional activity of PSII are disturbed, more O_2_^•−^, H_2_O_2_, or HO^•^ is produced in PSII [217,218].

Of the redox active components of PSII, the Pheos and Q_A_ and Q_B_ may be able to reduce O_2_. Formation of 21 µmol O_2_^•−^ (mg Chl)^−1^ h^−1^, measured as an SOD-dependent cytochrome *c* reduction, was observed in D1/D2/cytochrome b559 (cyt b559) complexes illuminated at 200 W m^−2^ in the presence of an artificial electron donor [217]. D1/D2/cyt b559 complexes lack Q_A_ and the Mn-cluster, and therefore the result suggests that Pheo_D1_^−^ (Pheo bound to the D1 protein) can be involved in the generation of O_2_^•−^. However, the reaction of O_2_ with Pheo_D1_^−^ is expected to be negligible in native RCs because of the very short lifetime of Pheo_D1_^−^ (Table 2). The redox potential of Pheo_D2_/Pheo_D2_^−^ (Pheo bound to the D2 protein) is 80–210 mV more negative than that of Pheo_D1_/Pheo_D1_^−^ [219] and therefore, if Pheo_D2_^−^ is formed, it would have a low enough redox potential to reduce O_2_.

It is difficult to tell whether Q_A_^−^ can reduce O_2_, as the *E_m_* of the pair O_2_/O_2_^•−^ depends on the hydrophobicity of the environment [12,54,58,59,60]. If the environment of O_2_ is equivalent to an aqueous solution, Q_A_^−^ would have a low enough potential to reduce O_2_, whereas the redox potential of O_2_/O_2_^•−^ in a hydrophobic environment is so low that Q_A_^−^ would be a poor reductant, although its lifetime is long enough for chemical reactions (Table 2). However, the participation of Q_A_^−^ in O_2_ reduction has been suggested [160,234,235,236]. O_2_^•−^ production was found to increase in the presence of an inhibitor of electron transfer at the Q_B_ site of PSII, DCMU (3-(3,4-di-chlorophenyl)-1,1-dimethyl urea), which was explained by the fact that DCMU increases the lifetime of Q_A_^−^ [236].

The production of O_2_^•−^ by both PSII with a functional Mn-cluster and by Ca^2+^ and Cl^−^-depleted PSII was detected using 5-diethoxyphosphoryl-5-methyl-1-pyrroline-*N*-oxide as a spin trap [235]. The generation of O_2_^•−^ at Q_A_ may occur due to the flexibility of the redox potential of Q_A_/Q_A_^−^, that has been reported to range from −80 to −200 mV (Table 2) and to depend on the structural and functional state of PSII. A shift in the redox potential of the Q_A_/Q_A_^−^ to a negative direction may cause the enhanced production of O_2_^•−^ in a PsbS (a chloroplast-localized protein required for NPQ) knock-out mutant [237].

Double-reduced Q_A_ has a very negative redox potential (Table 2) and can reduce O_2_. However, Q_A_^2−^ can only be formed by chemical treatment or by strong illumination in the absence of O_2_ [238]. Thus, the involvement of Q_A_^2−^ in O_2_ reduction seems unlikely.

**Figure 2:**
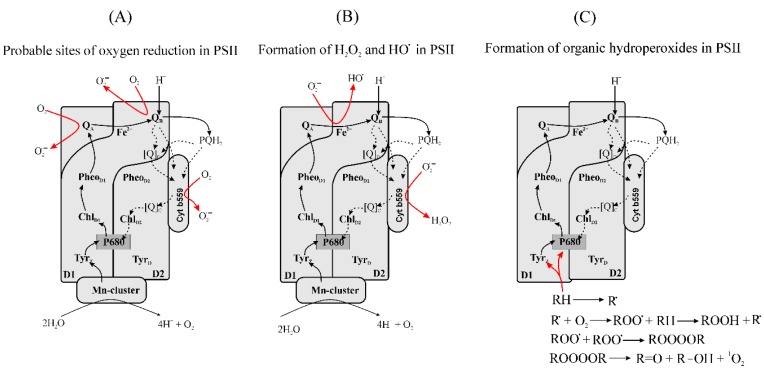
Formation of reactive oxygen species (ROS) in PSII. (**A**) Formation of superoxide (O_2_^•−^) can occur with the interaction of O_2_ with a semiquinone anion radicals at the Q_A_ and Q_B_ sites, when the electron flow from Q_B_ to the PQ pool is limited. The low potential form of cyt b559 can reduce O_2_ to O_2_^•−^ inside the thylakoid membrane [234,235,236]. (**B**) Formation of H_2_O_2_ and HO^•^. Cyt b559 can catalyze the formation of H_2_O_2_ inside the thylakoid membrane by a O_2_^•−^ dismutation mechanism [72,160]. O_2_^•−^ can reduce cyt b559 (Fe^3+^) to cyt b559 (Fe^2+^). O_2_^•−^ + cyt b559 (Fe^3+^) ∆ O_2_ + cyt b559 (Fe^2+^). The following interaction of HO_2_^•^ with Cyt b559 (Fe^2+^) leads to the formation of a ferric–hydroperoxo intermediate of cyt b559 (Fe^3+^–OOH) which can spontaneously decompose to cyt b559 (Fe^3+^) and H_2_O_2_. HO_2_^•^ + cyt b559 (Fe^2+^) → cyt b559 (Fe^3+^–OOH) + H^+^→ cyt b559 (Fe^3+^) + H_2_O_2._ The formation of H_2_O_2_ in a cyt b559-dependent way requires the protonation of O_2_^•−^ to form HO_2_^•^. The interaction of O_2_^•−^ with Fe^2+^ on the acceptor side of PSII can result in the formation of a ferric–peroxo intermediate [Fe^3+^–OO^−^] that can be protonated to a ferric–hydroperoxo intermediate [Fe^3+^–OOH]. O_2_^•−^ + [Fe^2+^] → [Fe^3+^–OO^−^] + H^+^ → [Fe^3+^–OOH]. [Fe^3+^–OOH] can be reduced by an electron from Q_A_^−^, which causes its decomposition to a ferric–oxo intermediate [Fe^3+^–O^−^] and HO^•^. Q_A_^−^ + [Fe^3+^–OOH] → Q_A_ + [Fe^3+^–O^−^] + HO^•^. (**C**) Formation of organic hydroperoxides on the donor side of PSII. Charge separation when the OEC is inactive leads to the formation of P680^•+^ and TyrZ^•^ which have a long lifetime and are therefore able to interact with surrounding molecules such as Chls, carotenoids and amino acids. The interaction of P680^•+^ or TyrZ ^•^ with an organic molecule (RH) can proceed via a radical chain mechanism [200,201,202].

Reduced Q_B_ is less likely to reduce O_2_ than Q_A_^−^ because the redox potential of Q_B_/Q_B_^−^ is around −45 mV (Table 2). Although Q_B_^−^ has a much longer lifetime than Q_A_^−^ when electron donation from Q_A_^−^ does not occur (Table 2), the quinone in the Q_B_ site is involved in proton-coupled electron transfer, and the redox potential of Q_B_^−^ becomes positive when the quinone is protonated (Table 2). The possible generation of O_2_^•−^ by quinones at Q_A_ and Q_B_ pockets is illustrated in Figure 2A.

In untreated chloroplasts, cyt b559 is found in high potential (HP), intermediate potential (IP) and low potential (LP) forms [239]. A very low potential (VLP) form was observed in isolated RCs of PSII [240] and seems to be an isolation artefact. The *E_m_* values of the three forms of cyt b559 are (see review [239]):

- HP form: 350–450 mV;
- IP form: 150–260 mV;
- LP form: −50–110 mV.

The ratio between the forms is variable and depends on isolation procedure. For example, modification of the donor side of PSII by removal of the Mn-cluster leads to the conversion of the HP form to the LP form [241]. In intact chloroplasts, the ratio of HP to LP forms was found to be 58 to 31, with respective redox potentials of 383 and 77 mV [242]. In untreated PSII membranes, the ratio HP:IP:LP was estimated as 44:31:25, with redox potentials of 375, 228 and 57 mV, respectively [243,244]. In isolated thylakoid membranes, 85% of cyt b559 was in the HP form [245]. The values measured from intact chloroplasts may best reflect the situation in vivo.

Cyt b559 has been suggested to be involved in cyclic electron transfer around PSII, where PQ bound in the specific binding pockets Q_D_ and Q_C_ acts as both an electron donor and an electron acceptor [239]. This idea is supported by the finding that PQH_2_ can reduce cyt b559 in both intact chloroplasts [246] and in PSII RC preparates [247]. The photoreduction of cyt b559 was found in isolated thylakoids and was inhibited by DCMU [248]. In Triton X-100-solubilized PSII particles, which mostly have the LP form of cyt b559, short-chain PQs stimulated both photoreduction and dark oxidation of cyt b559 [248].

The involvement of cyt b559 in electron transfer reactions of PSII indicates that cyt b559 is a redox active component that can potentially reduce O_2_. It has been shown that fast, dark reoxidation of the PQ pool in thylakoid membranes is not caused by direct oxidation of PQH_2_ by O_2_, and it was suggested that the LP form of cyt b559 can transfer an electron to O_2_ and thereby act as a PQH_2_:O_2_ oxidoreductase [248]. In isolated PSII membranes, O_2_ has been shown to compete with prenylquinones for oxidation of the LP form of cyt b559, suggesting that LP cyt b559 can form O_2_^•−^ [249]. Exogenously added short-chain quinones significantly enhance O_2_^•−^ production by PSII [245]. This finding was interpreted to indicate that these quinones reduce LP cyt b559, which then undergoes spontaneous autoxidation, resulting in O_2_^•−^ formation.

However, the reduction in O_2_ by LP cyt b559 is thermodynamically unfavorable, taking into account that the redox potential of the LP form is usually within 20–110 mV in untreated membranes, although sometimes an LP form with a negative potential is observed [239]. To explain O_2_ reduction via an apparently thermodynamically unlikely reaction, it has been suggested that the *E*_0_*′* of O_2_/O_2_^•−^ should be calculated by the Nernst equation, due to differences in concentrations of O_2_ and O_2_^•−^ [160]. According to the Nernst equation, and assuming concentrations of 250 µM and 500 nM for O_2_ and O_2_^•−^, respectively, the redox potential becomes close to 0 mV. Thus, the electron transfer from LP cyt 559 to O_2_ becomes feasible. However, it seems that the comparison of standard middle point potentials is more correct, as cyt b559 is bound within a protein matrix, and a considerable difference in the local concentrations of O_2_ and O_2_^•−^ is questionable.

A possible alternative solution is that cyt b559 mediates the formation of semiquinones at Q_D_ and Q_C_ sites. Experimental evidence of the reduction in O_2_ by a loosely bound plastosemiquinone anion radical (PQ^•−^) at the Q_C_ site was provided by Yadav et al. [250]. The authors showed that PQ^•−^ can be formed by a one electron reduction in PQ at the Q_B_ site and one electron oxidation of PQH_2_ by cyt b559 at the Q_C_ site. Because a PQ molecule has been crystallographically detected in the Q_C_ site [251], PQ might be tightly bound within the Q_C_ pocket and act as an electron carrier from cyt b559 to P680. The environment of the Q_D_ pocket is probably flexible and lipophilic and can facilitate a PQ/PQH_2_ exchange. In this case, the PQ pool can serve as an electron donor for cyt b559. It might be proposed that the formation of O_2_^•−^ in a cyt b559-dependent pathway couples cyt b559 and quinones and depends on the redox state of the PQ pool (Figure 2A). The rate constant of cyt b559-mediated reduction of O_2_ was estimated to be about 10^−6^ M^−1^ s^−1^ inside the thylakoid membrane, assuming that the reaction proceeds as a second-order chemical reaction [252].

The formation of O_2_^•−^ in PSII causes the formation of H_2_O_2_ via spontaneous dismutation (Reaction (5)) [234] or via a cyt b559-dependent catalytic reaction [253]. In isolated RCs of PSII, cyt b559 was found to exhibit SOD activity [217]. As proposed by Pospíšil [160], the catalytic formation of H_2_O_2_ by cyt b559 proceeds as a two-step reduction–oxidation reaction involving two molecules of O_2_^•−^. The first step is reduction of cyt b559 (Fe^3+^) to cyt b559 (Fe^2+^), Reaction (106). The second step is the oxidation of cyt b559 (Fe^2+^) by HO_2_^•^, the protonated form of O_2_^•−^, with formation of cyt b559 (Fe^3+^) and H_2_O_2_, Reaction (107).

O_2_^•−^ + cyt b559 (Fe^3+^) → O_2_ + cyt b559 (Fe^2+^) (106)

HO_2_^•^ + cyt b559 (Fe^2+^) → H_2_O_2_ + cyt b559 (Fe^3+^) (107)

According to this mechanism, the catalytic disproportionation of O_2_^•−^ should be pH-dependent because the protonated form of O_2_^•−^ is needed.

In addition to its formation by the dismutation of O_2_^•−^, H_2_O_2_ might also appear during incomplete oxidation of H_2_O by the Mn-cluster. It has been suggested that incomplete oxidation of H_2_O can occur during the two-electron oxidation of water by the Mn-cluster at the transition from the S_2_ state to the S_0_ state [234,254]. However, the two-electron oxidation of water during the transition of the Mn-cluster from S_3_ to S_1_ does not result in the formation of H_2_O_2_ [255].

In PSII, the Fenton mechanism involving a metal (M) cation, Reaction (58), can also function, leading to the formation of HO^•^.

In PSII, HO^•^ can be formed both in the dark and in the light. HO^•^ formation was shown when PSII membrane particles were heated in the dark [256]. The authors suggested that this process is associated with heat-induced changes of the PSII donor side and proceeds via the Fenton mechanism. The formation of HO^•^ was suppressed by CAT and metal chelators, indicating that the appearance of HO^•^ is related to the decomposition of H_2_O_2_. However, a high concentration of CAT, around 5000 U/mL, was required to suppress the appearance of HO^•^. Exogenous calcium and chloride prevented the appearance of HO^•^. Furthermore, no HO^•^-related EPR signal was observed after removal of the Mn-cluster by Tris-treatment of PSII membranes [257]. These data confirm that the Mn-cluster is likely involved in HO^•^ formation in PSII under heat stress in the dark.

The light-dependent formation of HO^•^ occurs in untreated PSII membranes [235,258,259,260]. Experimental results suggest that HO^•^ can be produced in the light by two pathways: firstly, by the well-known Fenton mechanism and secondly, by the reduction of a peroxide bound to the non-heme iron on the acceptor side of PSII [72]. The formation of HO^•^ at the non-heme iron is initiated by the binding of O_2_^•−^ and formation of an O_2_^•−^-iron complex that can be protonated to a ferric–hydroperoxo complex, Reactions (49) and (50). The ferric–hydroperoxo complex can be decomposed via reduction by Q_A_^−^ with the formation of HO^•^ and a ferric–oxo ((Fe^3+^)-O^−^) complex, Reaction (108).

L-(Fe^3+^)-OOH + Q_A_^−^ → L-(Fe^3+^)-O^−^ + HO^•^ + Q_A,_ (108)

where L is a ligand. Reaction (108) can be considered as a Fenton reaction proceeding with a bound hydroperoxide. The possible sites of formation in PSII are shown in Figure 2B.

The formation of bound hydroperoxides has been found to occur on the donor side of the PSII (Figure 2C). The mechanism is associated with the formation of a long-lived species, having a high positive redox potential in PSII. In PSII membranes holding an intact Mn-cluster, the O_2_ consumption rate is very low, around 1 μmol O_2_ (mg of Chl)^−1^ h^−1^ [128], but the rate becomes 6-fold higher in alkaline-treated and Mn-depleted PSII membranes [261]. O_2_ consumption was found to be associated, at least partially, with the generation of a component with positive charge(s) on the donor side of PSII, as the electron donors diphenylcarbazide and ferrocyanide suppressed the rate of O_2_ consumption caused by disruption of the donor side of PSII. A further study revealed that the removal of Mn from the OEC of PSII leads to O_2_ photoconsumption with a maximum at the first flash, with a yield comparable to the yield of O_2_ evolution on the third flash measured in the PSII samples before Mn removal [262]. Inactivation of the OEC can lead to the formation of both P680^•+^ and TyrZ^•^. In the absence of electron donation from the OEC, both will have a long lifetime, and will therefore be able to interact with surrounding molecules such as Chls, carotenoids and amino acids. Based on these results, it has been proposed that the formation of peroxides on the donor side of PSII proceeds via a radical chain mechanism, starting with P680^•+^ (or TyrZ^•^), Reactions (104), (105) and (109).

ROO^•^ + RH → ROOH + R^•^ (109)

The evidence of ROOH production on the donor side of PSII was obtained using the specific fluorescence probe SPY-HP [200]. In this work, highly lipophilic peroxides (LOOH) and relatively hydrophilic ones (ROOH), were distinguished by the rate of reaction with Spy-HP. The formation rates of both LOOH and ROOH were estimated to be 0.022 µmol LOOH (µmol RC)^−1^ s^−1^ and 1.11 µmol ROOH (µmol RC)^−1^ s^−1^, respectively [200]. The formation of carbon centred radicals, in turn, was found in PSII membranes with EPR spin-trapping technique when PSII membranes were treated by high light and heating. It has recently been found that exposure of Mn-depleted PSII membranes to high light results in the formation of protein radicals located mainly in the D1, D2, CP43 and CP47 proteins [202]. The formation of protein radicals is suppressed by diphenylcarbazide, indicating that protein radicals were formed by the oxidation of proteins by P680^•+^ or TyrZ^•^. The formation of protein radicals was correlated with the formation of hydroperoxides measured with the SPY-HP probe. The formation of R^•^ can initiate chain propagation reactions, and thereby lead to accumulation of ROOH (Reactions (105) and (109)).

#### 3.2.4. Formation of Reduced Forms of Oxygen, O_2_^•−^, H_2_O_2_, HO^•^, in PSI

The acceptor side of PSI is believed to be the predominant site of O_2_ reduction in thylakoid membranes, as O_2_ reduction depends on the PSI activity (see reviews [12,213,214,215]). It has been shown that both the photoreduction of cytochrome *c* and photooxidation of epinephrine, which have been used as traps for O_2_^•−^, were inhibited by SOD. This indicates that the reduction in O_2_ proceeds via univalent reduction, and O_2_^•−^ was identified as the primary product in illuminated thylakoids [124,263]. The predominant role of PSI in O_2_ reduction was shown in experiments with specific inhibitors that block PETC at different sites, and using a PSI-deficient mutant. The photoproduction of O_2_^•−^ in thylakoids is inhibited by DCMU and can be restored by the addition of AscH_2_ and *N*,*N*,*N*′,*N*′-tetramethyl-*p*-phenylenediamine, to provide electron donation to PC and P700, respectively [124,263,264]. This indicates that the contribution of PSII to the photoproduction of O_2_ in thylakoids is small_._ A slight influence of O_2_ on the steady-state level of Chl fluorescence in a PSI-deficient mutant of *Oenothera sp.* was attributed to insignificant leakage of electrons from PETC to O_2_, due to the suppression of Mehler’s reaction [265]. On the other hand, a significant rate of O_2_ reduction by thylakoids was observed in the presence of dibromothymoquinone (DBMIB) and dinitrophenylether of 2-iodo-4-nitrothymol (DNP-INT), inhibitors of PQH_2_ oxidation by Cyt b6f, [128,266]. It was found that the contribution of other sites of PETC, besides PSI, to O_2_ reduction increased with an increase in light intensity, and at high intensities achieved 60% of total O_2_ reduction in PETC. These data suggest that PSI is not the only site of O_2_ reduction in thylakoid membranes, but other sites of PETC can contribute to O_2_ reduction [128]. Thus, experiments with isolated PSI membranes can provide more correct measurements of activity of PSI in the photoproduction of O_2_^•−^.

**Figure 3:**
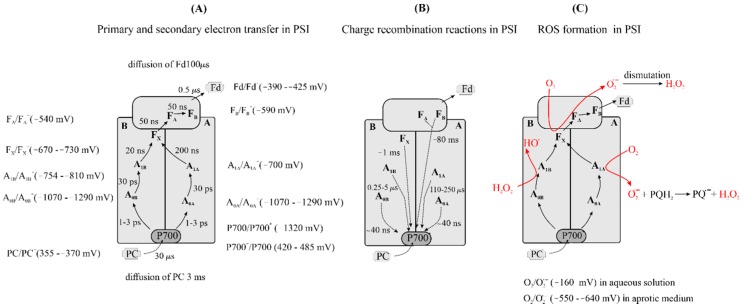
(**A**) Forward electron transfer chain, lifetimes and midpoint redox potentials of the cofactors of PSI; (**B**) charge recombination reactions and recombination lifetimes of the cofactors of PSI; the values were taken from [145,268,269,270,271]; (**C**) possible means of ROS formation in PSI [152,266,272,273,274,275]. PC is plastocyanin; P700 is a dimer of Chl *a* molecules, the primary electron donor; A_0A_ and A_0B_ are Chl *a* molecules located in branches A and B, respectively, both act as primary electron acceptors; A_1A_ and A_1B_ are phylloquinone molecules located in branch A and B, respectively, both acting as electron acceptors; F_X_, a 4Fe-4S cluster, a secondary electron acceptor; F_A_ and F_B_, 4Fe-4S clusters, terminal electron acceptors.

The electron transport chain within PSI (Figure 3) consists of two quasisymmetrical branches (A and B) containing six Chl, two phylloquinones (A_1_), and three 4Fe-4S clusters (F_X_, F_A_, and F_B_). Two Chl *a* molecules have been assigned to the spectroscopically characterized primary acceptor A_0_. Another pair of Chl *a* molecules is located between P700 and A_0_ and assigned as accessory Chls that may participate in excitation and/or electron transfer (for more details, see review [267]).

The mechanism of O_2_ reduction in PSI is still under debate. It was suggested that O_2_^•−^ production within the thylakoid membranes most likely occurs via autooxidation of the membrane-bound primary electron acceptors in PSI, possibly 4Fe-4S clusters (F_X_, F_A_, and F_B_) [152]. The *E_m_* of F_A_/F_A_^−^ and F_B_/F_B_^−^ and F_X_/F_X_^−^ vs. NHE were estimated to be −479, −539, and −650 mV, respectively (Figure 3) [145]. The reduction in O_2_ by F_X_ is thermodynamically favorable but kinetically less likely than a reduction in O_2_ by F_A_^−^ or F_B_^−^, as the lifetime of F_X_^−^ is less than 50 ns (Figure 3). When F_A_ and F_B_ clusters are reduced, the lifetime of F_X_ is limited by charge recombination [P700^+^F_X_^−^] and estimated to be ~250 µs [276].

Electron transfer from F_B_^−^ to Fd occurs within 1 µs, and therefore the oxidation of F_A_^−^ and F_B_^−^ by O_2_ in an aqueous region is not kinetically favorable in the presence of oxidized Fd (Figure 3). The lifetimes of F_A_^−^ and F_B_^−^ become much longer if Fd is mostly reduced or its diffusion to the F_A_ and F_B_ sites is limited. The charge recombination of [P700^+^F_A_^−^] and [P700^+^F_B_^−^] has a lifetime of about 50 ms when no extrinsic electron acceptors and donors are present [277]. The rate of O_2_^•−^ production by PSI in both isolated thylakoids and isolated PSI complexes ranges from 15 to 30 µmol O_2_^•−^ (mg Chl)^−1^ h^−1^, corresponding to 2.5–4.5 O_2_^•−^ per P700 s^−1^ if the ratio of P700 to total amount of Chl is 1 to 600 for isolated thylakoid membranes [124,264]. The rate of O_2_^•−^ production is at least one order of magnitude higher in PSI subchloroplast fragments in the presence of the surfactant Triton X-100 than in its absence [278]. The K_m_ value for O_2_ in photoreduction by PSI was estimated to be 2–3 µM in both thylakoid membranes and PSI subchloroplast fragments and the second order rate constant for O_2_ reduction by the electron acceptors of PSI was calculated to be 1.5 × 10^7^ M^−1^ s^−1^ [278]. In another work, the K_m_ value for O_2_ was estimated to equal to ~8 and ~3 µM for thylakoids, in the absence and in the presence of Triton X-100, respectively [279].

Experiments with O_2_^•−^-dependent protein iodination showed that O_2_^•−^ can also be produced in the aprotic interior of the thylakoid membrane close to the RC of PSI [272]. Thus, not only F_A_ and F_B_, but also F_X_ and A_1_, might be involved in O_2_^•−^ production within the thylakoid membrane. It has been suggested that O_2_^•−^ mediates cyclic electron transfer by donating electrons to Cyt b6f or to P700^+^, and this cycle would explain why the observed rate of O_2_^•−^ production is low in intact PSI [12]. The increase in O_2_^•−^ production in PSI subchloroplast fragments in the presence of Triton X-100 could result from the prevention of the putative O_2_^•−^ mediated cyclic electron flow around PSI, due to the disintegration of the supermolecular structure of PSI by Triton X-100 [12]. The increase in O_2_^•−^ production in the presence of ammonium ions, and amines is considered as evidence of an O_2_^•−^-mediated cyclic electron flow in PSI [272], as these substances supply protons to the membranes and accelerate the dismutation of O_2_^•−^. The dismutation of O_2_^•−^ prevents the O_2_^•−^-mediated cyclic electron flow, thereby increasing the detectable production of O_2_^•−^. Thus, dismutation of O_2_^•−^ in thylakoid membranes and cyclic electron flow in isolated PSI complexes would explain why the rate of O_2_^•−^ production is similar in these two preparations.

It has recently been suggested that the reduction in O_2_^•−^ by F_A_, F_B_ and F_X_ occurs in a lipophilic region [273]. As the dielectric constant in the immediate environment of the F_A_ and F_B_ centers is 5.4 [280], the redox potential of O_2_/O_2_^•−^ in aprotic medium (−550 and −600 mV vs. NHE [54] in DMF) should be used in comparisons of the redox potentials of PSI cofactors and O_2_/O_2_^•−^. Thus, the difference in the redox potentials of O_2_/O_2_^•−^ and PSI redox cofactors F_A_/F_A_^−^ and F_B_/F_B_^−^ would make the reduction of O_2_ by F_A_ and F_B_ thermodynamically less favorable. If we assume that O_2_^•−^ is produced via O_2_ reduction by F_A_ and F_B_ in a lipophilic environment, then the differences in redox potentials would easily explain why the rate of O_2_^•−^ production is low in PSI subchloroplast fragments. In this case, the effect of Triton X-100 would be to make the immediate area of F_A_ and F_B_ less lipophilic, which would shift the redox potential of O_2_/O_2_^•−^ toward positive values.

According to the *E_m_*, Fx and A_1_ would be favorable reductants of O_2_, even in an aprotic environment, as the *E_m_* values of the pairs A_1_/A_1_^−^ located on the A- and B-branches of PSI electron transfer chain are −0.7 and −0.81 mV, respectively (Figure 3). Indeed, phylloquinone A_1_ stimulated the flash-induced photoconsumption of O_2_ when added to thylakoid membranes from which A_1_ had been partially removed [266]. It was suggested that A_1_ could be the main reductant in O_2_^•−^ production in PSI. However, results regarding the importance of the phylloquinone in O_2_^•−^ production vary. Firstly, the stimulation of O_2_ photoconsumption by addition of A_1_ was observed only on the first flash [266]. The appearance of O_2_^•−^ on the outside and inside of thylakoid membranes was tested with hydrophilic and lipophilic cyclic hydroxylamines that react with O_2_^•−^, forming nitroxide radicals with a specific EPR spectrum [274]. In this work, a significant effect of SOD on the formation of both hydrophilic and lipophilic nitroxide radicals suggested that 90% of O_2_^•−^ is formed at the membrane surface or outside of the membrane. On the other hand, evidence of the participation of A_1_ in O_2_^•−^ formation was obtained with PSI complexes isolated from *menB* mutant, a phylloquinone-less knockout strain of the gene encoding 1,4-hydroxynaphthoyl-CoA-synthase of the cyanobacterium *Synechocystis* sp. PCC 6803. The mutant contains PQ at the phylloquinone-binding site A_1_ [275]. In the mutant, the redox potential of PQ bound to the A_1_ site was −553–−693 mV, close to the redox potential of F_X_/F_X_^−^ and about 100 mV more positive than that of A_1_/A_1_^−^ [281]. O_2_ photoconsumption in isolated PSI complexes of the mutant was found to be slower than in the wild type [275]. The low rate of O_2_ photoconsumption in the mutant was explained by the difference in the redox potentials of PQ and A_1_, and the results suggest that A_1_ is the main site of O_2_ reduction in PSI. *N*,*N*,*N*′,*N*′-Tetramethyl-*p*-phenylenediamine and AscH_2_ were used as electron donors.

A_1_^−^, located in the B-branch of PSI, decays within 20 ns by electron transfer to F_X_, whereas electron transfer from the A-branch A_1_^−^ takes 170 ns (Figure 3). A_1_^−^ can accumulate in high light when electron transfer from A_1_^−^ to F_X_ is limited. In this case, the electron flow from F_A_ and F_B_ to the MDA radical can prevent the accumulation of A_1_^−^, which minimizes the interaction of O_2_ with A_1_^−^. MDA is formed mainly by a reaction between APX and AscH_2_ (Reactions (62)–(64)). MDA can also be formed by a reaction between AscH_2_ and O_2_^•−^, and MDA is an effective electron acceptor of PSI, effectively competing with methyl viologen [282]. The reduction in MDA by PSI occurs via reduced Fd [283]. In summary, a number of AscH_2_-related reactions can influence the photoreduction of O_2_ by PSI (Reactions (34), (62)–(64) and (110)–(113)), and the large number of reactions and reactants makes it difficult to estimate the importance of AscH_2_/MDA/DHA in O_2_ reduction in PSI.

PSI^red^ + O_2_ → PSI^ox^ + O_2_^•−^ (110)

O_2_^•−^ + AscH_2_ → H_2_O_2_ + MDA (111)

PSI^red^ + MDA + H^+^ → PSI^ox^ + AscH^−^ (112)

MDA + MDA + 2H^+^ → AscH_2_ + DHA (113)

From data presented by Kozuleva et al. [275], the rate of O_2_^•−^ production by PSI can be estimated to be 2.5 O_2_^•−^ per P700 s^−1^ according to the O_2_ consumption rate (250 µmol O_2_ (mg Chl)^−1^ h^−1^) assuming 40 molecules of Chl per P700 in isolated PSI complexes [278]. If the production of O_2_^•−^ by PSI proceeds as an elementary second-order reaction, then the second-order rate constant is about 10^4^ M^−1^ s^−1^ for a saturated concentration of O_2_ in aqueous solution. 4Fe-4S clusters of PSI can have a low efficiency toward O_2_ reduction, and the second-order rate constant of the reaction of O_2_ with Fe-S proteins like Fd is about 10^3^ M^−1^ s^−1^ [121]. However, the rate of O_2_ reduction by PSI was saturated to above 20 µM of O_2_, with the second-order rate constant 1.5 × 10^7^ M^−1^ s^−1^ at a high light intensity [278]. The reaction of O_2_ with semiquinones having low redox potential proceeds with rate constants in the range of 10^8^–10^9^ M^−1^ s^−1^ [61]. These data may suggest that cooperation between 4Fe-4S clusters and phylloquinones A_1_ can provide flexibility for the O_2_^•−^ formation inside and outside of the thylakoid membrane. In high light, O_2_^•−^ formation by A_1_ becomes more important, which leads to the accumulation of O_2_^•−^ within the membrane.

In the aqueous phase, the dismutation of O_2_^•−^ is catalyzed by SOD (Reaction (5)). Intramembranous formation of O_2_^•−^, in turn, can lead to the formation of H_2_O_2_ within the thylakoid membrane due to the reaction of O_2_^•−^ with PQH_2_, Reaction (114) [128,264,266].

PQH_2_ + O_2_^•−^ → PQ^•−^ + H_2_O_2_ (114)

The second-order rate constant for the reaction of O_2_^•−^ with PQH_2_ was estimated to be 4 × 10^4^ M^−1^ s^−1^ in acetonitrile [252]. The accumulation of H_2_O_2_ in a thylakoid suspension in the presence of cytochrome *c*, that reacts with O_2_^•−^ in aqueous phase (Reaction (31)) preventing the dismutation of O_2_^•−^, was attributed to the formation of H_2_O_2_ within the thylakoid membrane via Reaction (114) [284].

The formation of H_2_O_2_ may contribute to the production of HO^•^ near PSI via the Fenton and Haber-Weiss reactions (Reactions (3) and (58)).

It was shown that the formation of HO^•^ in broken chloroplasts was suppressed by DCMU and it was suggested that HO^•^ is predominantly produced in PSI via the reduction in H_2_O_2_ by protein-bound iron in PSI, as the metal chelator Desferal did not suppress HO^•^ production [157]. The formation of HO^•^ via the reaction of O_2_^•−^ with the terminal acceptors F_X_, F_A_, and F_B_ of PSI was recently suggested [285]. However, this route of HO^•^ formation requires the dismutation of O_2_^•−^ to form H_2_O_2_ as an intermediate. In the presence of PQH_2_, the production of O_2_^•−^ would lead to the accumulation of H_2_O_2_ within the thylakoid membrane. The redox potential of H_2_O_2_/(HO^•^, ^−^OH) is 400–600 mV in organic solvents [286], and therefore the presence of H_2_O_2_ in the vicinity of the A_1_ site, with a much more negative redox potential (Figure 3), would lead to the formation of HO^•^ in a Fenton-type reaction of H_2_O_2_ with phylloquinone A_1_^•−^ (Reaction (115)) [252].

A_1_^•−^ + H_2_O_2_ → A_1_+ HO^•^ + ^−^OH (115)

#### 3.2.5. Formation of Reduced Forms of Oxygen, O_2_^•−^, H_2_O_2_, HO^•^, in the PQ Pool and by Cyt b6f

PQ is a prenyllipid consisting of 2,3-dimethyl-1,4-benzoquinone and a side chain of nine isoprenyl units attached to Position 5. The total amount of PQ in leaves is in the range 25–40 molecules per P700 [248,287,288,289]. PQ has been found in thylakoid membranes, the envelope of the chloroplast and plastoglobules. [290,291,292,293,294]. The ratio of PQ in the envelope and PQ in the thylakoid membrane was found to be 2:5 [293]. The PQ involved in electron transfer in the thylakoid membrane is called the photoactive PQ and its amount is in the range 6–15 PQ per P700, assuming that the ratio of P700 and Chl is 1/600 [248,288,295,296,297,298]. PQ can be present in three forms: PQ, PQ^•−^, and PQH_2_. Both reduced forms can exist in protonated and deprotonated forms: PQH^•^or PQ^•−^, and PQH_2_, PQH^−^ or PQ^2−^. The pK_1_ and pK_2_ values of PQH_2_ in aqueous solutions are 10.8 and 12.9; the pKa value of PQH^•^ is 5.9 [299]. The above data were measured for plastoquinone-1, which has only one prenyl group attached to Position 5 of 2,3-dimethyl-1,4-benzoquinone.

**Figure 4:**
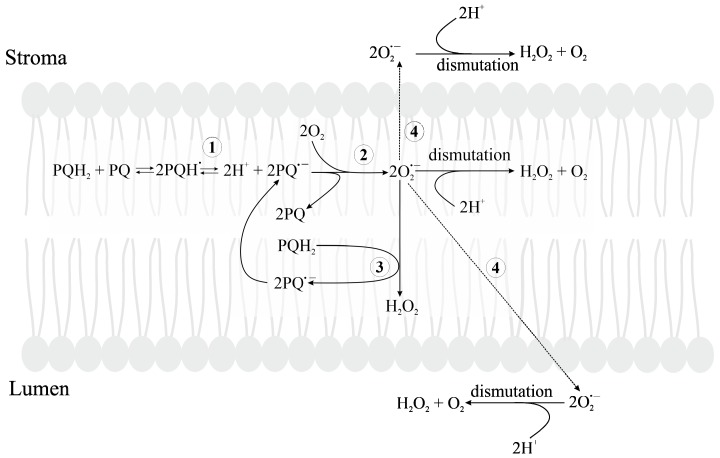
Autocatalytic oxidation of reduced plastoquinone (PQH_2_) by O_2_ in the thylakoid membrane. 1—formation of a plastosemiquinone radical (PQH^•^) by a dismutation reaction of PQH_2_ with PQ; 2—reduction in O_2_ by a plastosemiquinone anion radical (PQ^•−^) with formation of superoxide anion radical (O_2_^•−^); 3—oxidation of PQH_2_ by O_2_^•−^ with formation of hydrogen peroxide (H_2_O_2_) and PQ; 4—diffusion of O_2_^•−^ from thylakoid membrane to stroma and to lumen. In the autocatalytic oxidation of PQH_2_, the reaction of O_2_^•−^ with PQH_2_ provides excess PQ^•−^ that can be involved in the formation of O_2_^•−^ and, in turn, accelerates the oxidation of PQH_2_ [128].

Significant PSI-independent O_2_ reduction was observed in a thylakoid suspension in the presence of the DNP-INT, that prevents the oxidation of PQH_2_ by Cyt b6f [128,236,266]. In the work of Kruk et al. [266], significant O_2_ reduction was also demonstrated in the presence of DBMIB, another inhibitor of oxidation of PQH_2_ by the Cyt b6f. DBMIB was found to strongly inhibit O_2_^•−^ production, whereas the formation of H_2_O_2_ was only partially inhibited. Furthermore, the rate of H_2_O_2_ production increased with the concentration of DBMIB [300]. On other hand, the removal, by a repeated freeze-thaw procedure, of PC, suppressed O_2_ reduction by thylakoid membranes. In addition, the PC-inhibitor HgCl_2_ significantly suppressed O_2_ reduction [266]. These data may suggest that the suppression of PSI-independent O_2_ reduction requires a strong inhibition procedure that may cause unspecific damage to the photosynthetic apparatus. In the work of Cleland and Grace [236], the production of O_2_^•−^ in the presence of DNP-INT was attributed to O_2_ reduction by Q_A_^−^. However, Khorobrykh and Ivanov [128] showed that PSI-independent O_2_ consumption in thylakoids was suppressed by DCMU, and O_2_ consumption by isolated PSII membranes was low. Thus, PSI-independent O_2_ consumption in thylakoid membranes in the presence of DNP-INT was interpreted as O_2_ reduction occurring in the PQ pool. The amount of detectable O_2_^•−^, measured using cytochrome c as a trap of O_2_^•−^, was found to be about 25% of the amount of O_2_^•−^ estimated from the O_2_ consumption rate [128]. This indicates that O_2_ reduction occurs mainly inside the thylakoid membrane, where O_2_^•−^ can be consumed in concomitant reactions. It has been proposed that O_2_ reduction in the PQ pool develops as a two-stage autocatalytic process that starts by the production of PQH^•^ via dismutation (Reaction (116)) and is followed by the deprotonation of PQH^•^ and subsequent oxidation of PQ^•−^ by O_2_ with the formation of O_2_^•−^ and PQ. Furthermore, PQH_2_ can be oxidized by O_2_^•−^ with the formation of PQ^•−^ that would again react with O_2_ to produce O_2_^•−^ and PQ (Figure 4) [128].

Thylakoid membranes have also been shown to accumulate H_2_O_2_ in the presence of cytochrome *c* that reacts with O_2_^•−^ and prevents the formation of H_2_O_2_ via superoxide dismutation (Reaction (31)) [284]. These data suggest that a considerable amount of H_2_O_2_ is generated inside the thylakoid membrane in the reaction of O_2_^•−^ with PQH_2_ ([284], Figure 4), as earlier suggested by Khorobrykh and Ivanov [128]. These results contradict with the results of Asada et al. [124], where cytochrome *c* completely inhibited H_2_O_2_ formation by thylakoids. However, in a later work of Takahashi and Asada [272], the formation of H_2_O_2_ in the presence of cytochrome *c* was shown. It is possible that different light intensities caused the contradiction, as H_2_O_2_ formation appears to increase with light intensity [284].

The mechanism and efficiency of O_2_ reduction in the PQ pool are under debate. Autooxidation of PQH_2_ is one possible mechanism (Figure 4) but is it biologically significant? According to the redox potential, the reduction in O_2_ by both PQ^•−^ and PQ^2−^ is thermodynamically favorable in aqueous solution since the redox potentials of PQ/PQ^•−^ and PQ^•−^/PQ^2−^ are −165 and −274 mV, respectively [299]. The deprotonation of PQH_2_ or PQH^•^ is essential for O_2_ reduction. Since PQ^2−^ is mostly protonated under physiological pH, PQ^•−^ was considered the main form of reduced PQ that could be involved in O_2_ reduction in thylakoids. The reactions of semiquinones with O_2_ with formation of O_2_^•−^ are equilibrium reactions where the quinone can be reduced by O_2_^•−^ (Reaction (31)).

The equilibrium constant for the reaction of O_2_ with PQ^•−^, as determined by the equation (RT/F)lnK = E(O_2_/O_2_^•−^) − E(Q/Q^•−^), where R is the gas constant, T is temperature and K is the equilibrium constant, and F is the Faraday constant, is 1.56 if the redox potentials of PQ/PQ^•−^ and O_2_/O_2_^•−^ are −165 and −160 mV, respectively [61]. The forward and reverse second-order rate constants for the formation of O_2_^•−^ by PQ^•−^ (Reaction (31)) are *k_forward_* ~ 10^8^ M^−1^ s^−1^ and *k_reverse_* ~ 7 × 10^7^ M^−1^ s^−1^ [61]. The equilibrium constant for Reaction (116) was estimated to be 10^−9.2^ [301], which shows that the formation of PQH^•^ via Reaction (116) is negligible. Thus, the apparent rate of O_2_^•−^ production in the PQ pool is determined by the rate of PQ^•−^ appearance, rate of O_2_^•−^ production via reaction of O_2_ with PQ^•−^ (Reaction (31)) and the rate of O_2_^•−^ removal from the equilibrium Reaction (31). In solvents with pure of PQH_2_ and PQ, the apparent rate of O_2_^•−^ production obviously results from the following reactions:

PQH_2_ + PQ ∆ 2PQH^•^ (116)

PQ^•−^ + H^+^ ∆ PQH^•^ (117)

Q^•−^ + O_2_ ∆ Q + O_2_^•−^ Reaction (31)

PQH_2_ + O_2_^•−^ → PQ^•−^ + H_2_O_2_ Reaction (114)

O_2_^•−^ + O_2_^•−^ + 2H^+^ → H_2_O_2_ + (O_2_ or ^1^O_2_) Reaction (5)

In thylakoid membranes, PQ is reduced by PSII to PQH_2_ in the light. This lowers the concentration of PQ, thereby preventing reaction (31) and leading to an increase in PQH_2_ oxidation by O_2_^•−^.

In organic solvents, the redox potentials of both PQ/PQ^•−^ and O_2_/O_2_^•−^ become more negative. The redox potentials of PQ/PQ^•−^ and O_2_/O_2_^•−^ were estimated to be −400 [302,303] and −600 mV vs. NHE in DMF [54] and around −640 mV in acetonitrile [59,60], respectively. According to the redox potentials, the equilibrium constant is 10^−7.8^, and therefore a reaction of PQ^•−^ with O_2_ is thermodynamically unfavorable in an aprotic medium. Thus, efficient O_2_^•−^ production via Reaction (31) can be observed only in an aqueous solution or at the membrane–water interface, or in a protein pocket where the redox potentials of O_2_/O_2_^•−^ and PQ/PQ^•−^ can be equal. The second-order rate constants for the autoxidation of PQH_2_ in different solvents, estimated from the initial rates, were found to range from 10^−2^ to 10^−3^ M^−1^ s^−1^ for both aqueous and aprotic solvents [248,252]. However, fast PQH_2_ oxidation in organic solvent was observed after the addition of KOH [252]. This reaction likely results from the formation of PQ^2−^ with a very negative redox potential, −1.1 V for PQ/PQ^2−^ [301]. The rate constant of PQH_2_ oxidation associated with O_2_ reduction by the PQ pool in thylakoids was estimated to be ~10^3^ M^−1^ s^−1^ if the reaction occurs inside the thylakoid membrane [128], and a later work calculated this rate constant to be 1.21 × 10^−3^ M s^−1^ while the rate of PQH_2_ autoxidation was ~10^−8^ M s^−1^ [252]. These rates were calculated assuming that PQH_2_ oxidation by O_2_ is a second-order chemical reaction and the oxidation of PQH_2_ occurs in the volume of thylakoid membrane. The steady-state concentration of PQ^•−^ inside the thylakoid membrane produced via reaction (116) can be estimated to be about 10^−8^ M. The following values were used in the calculations: amount of photoactive PQ, 14 × 10^−3^ mol PQ (mol Chl)^−1^ [248]; volume of thylakoid membrane, 4.6 × 10^−6^ L (mg Chl)^−1^ [304]; molar mass of Chl, 894 g mol^−1^ [304]; the equilibrium constant for reaction (116) was taken as 10^−10^; and the ratio of PQ and PQH_2_ was taken as 1/9. If O_2_^•−^ is very rapidly removed and therefore Reaction (31) can be considered an irreversible reaction with a second order rate constant of about 10^8^ M^−1^ s^−1^, then the rate of PQH_2_ oxidation by O_2_ inside the thylakoid membrane can be estimated to be 2.4 × 10^−3^ M s^−1^. This estimated rate is close to the rate of PQH_2_ oxidation by O_2_ inside the thylakoid membrane, calculated from the O_2_ consumption rate [252]. Thus, O_2_ reduction by the PQ pool via an autoxidation mechanism (Figure 4) occurs when O_2_^•−^ is efficiently consumed and the second-order rate constant is about 10^8^ M^−1^ s^−1^. The consumption of O_2_^•−^ can occur via the reaction of O_2_^•−^ with PQH_2_, Reaction (114). The second-order rate constant for the reaction of O_2_^•−^ with PQH_2_ has recently been estimated to be 4 × 10^4^ M^−1^ s^−1^ [252]. The second-order rate constant for the oxidation of PQH_2_ by O_2_ in illuminated thylakoids is within 10^2^–10^3^ M^−1^ s^−1^. Thus, the autoxidation of PQH_2_ by O_2_ can explain O_2_ consumption in the light in the presence of an inhibitor of Cyt b6f only with some assumptions. In addition, the rate of PQH_2_ oxidation in thylakoids in the light is over 20 times as fast as the rate of oxidation of PQH_2_ in the dark after photoreduction [248], suggesting that light-dependent reaction(s) dominate in the O_2_-dependent oxidation of the PQ pool.

The PQ pool has also been found to scavenge ^1^O_2_ in thylakoid membranes [305,306,307], with the second-order rate constant of the reaction of ^1^O_2_ with PQH_2_, 0.97 × 10^8^ M^−1^ s^−1^ in acetonitrile [50]. The reaction of ^1^O_2_ with PQH_2_ in methanol was found to lead to the formation of H_2_O_2_ (Reaction (119)) [48]. It was suggested that the reaction of ^1^O_2_ with PQH_2_ is initiated by the formation of ^1^O_2_ in PSII and can also proceed inside the thylakoid membrane [48]. The formation of H_2_O_2_ via oxidation of PQH_2_ by ^1^O_2_ may occur in two ways. In the first one, ^1^O_2_ reacts with PQH_2_ to form an unstable hydroperoxide adduct of the quinone ring (PQH_2_-OO), which directly decomposes to form H_2_O_2_ and PQ (Reaction (119)). In the second way, the hydroperoxide adduct decomposes to form HO_2_^•^ and PQH^•^ (Reaction (120)). This indirect mechanism would be similar to that proposed for the oxidation of AscH_2_ by ^1^O_2_ [47]. In the indirect mechanism, H_2_O_2_ is produced by the oxidation of PQH_2_ to PQH^•^ by HO_2_^•^ (Reaction (121)).

^3^P680 + O_2_ → P680 + ^1^O_2_ (118)

^1^O_2_ + PQH_2_ → [PQH_2_-OO] → PQ + H_2_O_2_ (119)

^1^O_2_ + PQH_2_ → [PQH_2_-OO] → PQH^•^ + HO_2_^•^ (120)

PQH_2_ + HO_2_^•^ → PQH^•^ + H_2_O_2_ (121)

Thus, the PSI-independent O_2_ reduction in the PQ pool may depend on ^1^O_2_ production in PSII, and this reaction can cause the formation of H_2_O_2_ inside the thylakoid membrane.

O_2_ reduction, associated with the PQ pool, also occurs without any inhibitors [264]. The ratio of the rate of O_2_ reduction in PSI and the rate of O_2_ reduction in the PQ pool, in the absence of any inhibitors, reaches 1:1 at a high light intensity [264]. However, the rate of O_2_ reduction by the PQ pool in the presence of DNP-INT is saturated at a low light intensity [128,264,308]. These data confirm that O_2_ reduction in a PQ pool in thylakoids without any inhibitors can occur parallel to O_2_ reduction in PSI. Interestingly, efficient O_2_ reduction by the PQ pool is observed at pH 5.0 in the absence of inhibitors, but not in the presence of DNP-INT [128,264]. The simplest explanation for differences in O_2_ reduction by the PQ pool in the absence and presence of DNP-INT, is to assume that the formation of O_2_^•−^ occurs in PSI. O_2_^•−^ can react with PQH_2_ to form H_2_O_2_ via Reaction (114) [264].

The autoxidation of PQH_2_ does not imply the participation of any enzymes. However, the oxidation of the PQ pool with PTOX is widely discussed [309]. PTOX is a non-heme diiron quinol oxidase that oxidizes PQH_2_ and reduces O_2_ to H_2_O. PTOX is localized in the non-appressed regions of the thylakoid membrane [310]. It has been suggested that PTOX provides an alternative electron flow from the PQ pool to O_2_ to prevent photoinhibition of PSII [311]. However, in higher plants, PTOX-mediated electron flow to O_2_ is negligible [312] or its contribution is less than one percent of the total electron flow through PETC [313,314]. However, PTOX may depend on conditions, as high PTOX content and high PTOX activity were induced in the alpine species *Ranunculus glacialis* L. during growth in strong light [315]. The rate of PTOX-mediated electron flow is approximately 0.3 e^−^ s^−1^ (P680)^−1^ [313]. This makes the rate of PQH_2_ oxidation equal to 0.15 PQH_2_ (P680)^−1^ s^−1^, or 0.35 μmol PQH_2_ (mg Chl)^−1^ h^−1^, assuming that the ratio of PSII to Chl is 1:420. Thus, the rate of PQH_2_ oxidation can be estimated to be 8.68 × 10^−5^ M s^−1^ inside the thylakoid membrane [252]. The second-order rate constant of PTOX-mediated oxidation of PQH_2_ inside the thylakoid membrane is 10.6 M^−1^ s^−1^. In the light, the oxidation of PQH_2_ by PTOX, associated with the reduction of O_2_ to H_2_O, would not lead to consumption of O_2_ because of its matching stoichiometry with O_2_ production by PSII, Reactions (122) and (123).

2PQH_2_ + O_2_ → 2PQ + 2H_2_O (122)

PSII + 2H_2_O + 2PQ → 2PQH_2_ + O_2_ (123)

PTOX-mediated electron flow to O_2_ is assumed to produce no ROS. However, isolated PTOX can oxidize decylPQH_2_ with the formation of O_2_^•−^ or H_2_O_2_ at pH 8.0 or in substrate-limiting concentrations [316]. The efficiency of ROS production by PTOX was estimated to be around 17% of the total O_2_-reduction activity of PTOX [316]. The rate of PTOX-mediated PQH_2_ oxidation associated with the formation of H_2_O_2_ was estimated to be 1.47 × 10^−5^ M s^−1^, with the second-order rate constant of 1.8 M^−1^ s^−1^ inside the thylakoid membrane [252]. Thus, the estimated rate of PTOX-mediated O_2_ reduction is 100 times less than the rate of O_2_ reduction by the PQ pool in illuminated thylakoids. Furthermore, if O_2_^•−^ is formed by PTOX, PQ^•−^ might also be formed.

PQ^•−^ is also considered a source of O_2_^•−^ production by Cyt b6f via the reaction of O_2_ with PQ^•−^. It has been suggested that PQ^•−^, generated via one-electron oxidation of PQH_2_ at the Q_O_ site by the 2Fe-2S cluster of the high-potential, Rieske iron–sulfur protein of the Cyt b6f (Reaction (124)), can be oxidized by the conversion of O_2_ to O_2_^•−^ [317].

PQH_2_ + 2Fe-2S_ox_ → PQ^•−^ + 2Fe-2S_red_ + 2H^+^ (124)

**Figure 5:**
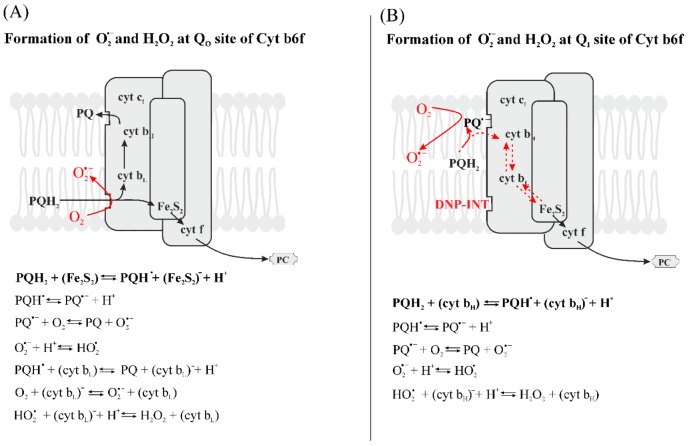
The proposed generation of O_2_^•−^ in Cyt b6f. (**A**) In the Q_O_ site of Cyt b6f, PQH^•^ is generated (Table 2) by the 2Fe-2S cluster of the high-potential Rieske iron−sulfur protein. PQ^•−^ that has a long residence time within the Q_O_ pocket, and cytochrome *b*_L_ can also serve as a reductant for the generation of O_2_^•−^ in Cyt b6f. In addition, HO_2_^•^, can be reduced by cytochrome *b*_L_ to form H_2_O_2_ [317]. (**B**) The proposed generation of O_2_^•−^ in Cyt b6f in the presence of DNP-INT, an inhibitor of PQH_2_ oxidation by Cyt b6f. The oxidation of PQH_2_ does not occur in Q_O_ site, formation of PQH^•^ and its deprotonation can occur in the Qi site. PQH^•^ can be oxidized in subsequent reactions with O_2_ or with hemes *b*_H_ or *b*_L_. H_2_O_2_ can be formed via the reaction of O_2_^•−^ with PQH_2_ or via the reaction of HO_2_^•^ with cytochrome *b*_H_ or cytochrome *b*_L_.

Isolated Cyt b6f complexes have been shown to produce H_2_O_2_ when decylPQH_2_ and PC were used as an electron donor and electron acceptor, respectively [317]. It was suggested that H_2_O_2_ appeared via O_2_^•−^ dismutation. No detectable O_2_^•−^ formation is observed in the presence of DBMIB, which has been shown to bind to an iron-sulfur binding site and at a position distal to the iron–sulfur binding site in Cyt b6f. This indicates that the mechanism of H_2_O_2_ production is related to the oxidation of PQH_2_ at the Q_O_ site of Cyt b6f. The production of O_2_^•−^ in Cyt b6f was also shown with EPR spectroscopy [317]. O_2_^•−^ can be formed via the interaction of O_2_ with PQ^•−^ in the Q_O_ pocket or/and with the interaction of O_2_ with the reduced form of p-side heme b_p_ [317]. The rate of O_2_^•−^ production by the isolated Cyt b6f is 4.5 (Cyt b6f)^−1^ s^−1^. This gives a rate of O_2_^•−^ production inside thylakoid membrane of about 2.6 × 10^−3^ M s^−1^, assuming that the ratio of Cyt b6f and Chl is 1:420 and the volume of the thylakoid membrane is 4.6 × 10^−6^ L (mg Chl)^−1^ [304]. This rate is close to the rate of O_2_^•−^ production by the PQ pool in the presence of DNP-INT. As DNP-INT blocks the oxidation of PQH_2_ at the Q_O_ site, the oxidation of PQH_2_ in the Q_O_ site cannot be responsible for O_2_ reduction in the PQ pool in the presence of DNP-INT. The formation of PQ^•−^ at the Qi site of Cyt b6f appears to cause O_2_ reduction in the PQ pool in the presence of DNP-INT (S. Khorobrykh and E. Tyystjärvi, unpublished data). The possible means of the reduction in O_2_ in Cyt b6f are shown in Figure 5.

The formation of HO^•^ has never been detected in the PQ pool, although it is supposed to happen. In the chloroplast stroma, H_2_O_2_ is efficiently scavenged, which would limit HO^•^ formation. However, H_2_O_2_ formed inside membranes by the PQ pool is not efficiently scavenged, and may therefore react with PQ^•−^ to form HO^•^ via the Fenton mechanism (Reaction (125)).

PQ^•−^ + H_2_O_2_ → PQ + HO^•^ + OH^−^ (125)

## 4. Damage Caused by ROS in the Chloroplast

### 4.1. Damage to PSII

PSII is the main producer of ^1^O_2_ in the chloroplast and a minor producer of other ROS (see Section 3.2), and therefore it is of great interest whether PSII is damaged by ^1^O_2_. In isolated PSII core complexes, electron transfer activity is lost and pigments are bleached only in the presence of O_2,_ suggesting an effect of ROS [318]. Furthermore, PSII is sensitive to damage caused by externally applied ^1^O_2_, as shown by a decrease in the quantum yield of PSII in lincomycin-treated tobacco leaves illuminated with the ^1^O_2_ sensitizer Rose Bengal [189].

The above results indicate that PSII can be damaged by ^1^O_2_ but do not prove that ^1^O_2_ produced by PSII is the agent of damage in the photoinhibition of PSII (for reviews, see [319,320]). The photoinhibition of thylakoid membranes does not depend on O_2_ and has a similar action spectrum under aerobic and anaerobic conditions [321], and photoinhibition in lincomycin-treated spinach leaf disks is only slightly slower in CO_2_ doped N_2_ than in air [322]. The effects of both deuterium oxide and ROS scavengers (reviewed by [319]) are variable and may depend on the type of complex. Similarly, effects of intrinsic ^1^O_2_ quenchers and scavengers vary, as overproduction of the xanthophyll zeaxanthin protects against photoinhibition in vivo in the green alga *Chlamydomonas reinhardtii* [323] and the carotenoid-rich mutant ΔSigCDE of the cyanobacterium *Synechocystis* sp. PCC 6803 show protection against the damaging reaction of photoinhibition [193], whereas the same reaction is not more rapid in α-tocopherol-deficient mutants of *Arabidopsis* [324] and *Synechocystis* [325]. Further indirect evidence on the participation of ^1^O_2_ in photoinhibition of PSII also varies, as the modification of the recombination reactions of PSII toward non-^1^O_2_-producing direction provides protection against photoinhibition [326], whereas the protection offered by NPQ is very limited [327], suggesting that the photoinhibition of PSII may not depend only on the excitation of Chl [320,328]. The photoinhibition-tolerant green alga *Chlorella ohadii* exhibits a recombination reaction model that is expected to lead to low ^1^O_2_ production [329].

Apart from their direct effect on PSII electron transfer activity, ROS have been shown to cause loss [318] and fragmentation [330,331,332,333] of the D1 protein in isolated PSII core complexes [330] and PSII membranes [333]. Both ^1^O_2_ [330] and H_2_O_2_ [332] cause fragmentation of the D1 protein. Miyao [333] concluded, on the basis of the protective effects of ROS scavengers, that several ROS, including O_2_^•−^, H_2_O_2_, ^1^O_2_ and HO^•^, participate in protein damage in PSII. The connection of these results to what happens in vivo is unclear, as the proteases responsible for the degradation of the D1 protein in vivo (for review, see [334]) have not been shown to be present in isolated PSII preparations. Kale et al. [335] detected the formation of O_2_^•−^ and HO^•^ in illuminated PSII membranes. Furthermore, the oxidative modifications of several amino acid residues of the D1 and D2 proteins were found to be associated with the formation of the radicals. Interestingly, both radicals were formed by PSII membranes throughout the illumination period, suggesting that they could contribute to the O_2_-dependent part of photoinhibition of PSII.

### 4.2. Damage to PSI

PSI has long been known to become inhibited, at least in certain plants, at chilling temperatures [336], in a reaction that depends on electron transfer from PSII to PSI [337]. The damage targets the iron–sulfur centers of PSI, and the remaining inactive PSI still functions as an excitation energy quencher [338]. The dependence of the photoinhibition of PSI on electron transfer, and the ability of PSI to reduce O_2_ to O_2_^•−^, strongly suggest that O_2_^•−^, H_2_O_2_ or HO^•^ participate in the damage [339]. However, neither the identity of the inhibitory ROS nor the exact site and the mechanism of production are known. Damage to PSI can be specifically induced by the application of fluctuating light, either in the form of short (10–300 ms) strong flashes [340], or in the form of few-seconds-long, saturating but not very strong flashes fired on top of short-term exposure of the plant to weak, PSII-specific light [341].

### 4.3. Oxidation of Membrane Lipids by ROS

Unsaturated fatty acids of membrane lipids can become peroxidated in a reaction with ^1^O_2_ (Reaction (20)) or HO^•^ (reaction (77)). Peroxidation by ^1^O_2_ dominates the non-enzymatic formation of lipid peroxides in leaves, whereas radical-induced peroxidation is more common in non-photosynthetic tissues [4].

Fatty acid peroxides, in turn, decompose either spontaneously or enzymatically to oxylipin carbonyls [342]. Tri-unsaturated fatty acids especially fragment to malondialdehyde that is highly reactive in its protonated dialdehyde form (O=CH-CH2-CH=O) [343]. Both malondealdehyde and acrolein, another highly reactive fragmentation product, are produced under non-stressed conditions but their concentrations increase during stress [344,345]. Lipid–peroxide-derived aldehydes and ketones like malondialdehyde function both as agents of damage and signaling molecules in *Arabidopsis* [344,346]. Due to their reactivity towards ROS, tri-unsaturated fatty acids may function as ROS sinks [347], and signaling by products of lipid oxidation may be essential for plant cells’ ability to survive oxidative stress [346].

### 4.4. Damage to Stromal Proteins

The production of ROS in the chloroplast is expected to damage proteins of the compartment of origin. In thylakoid membranes, light-induced damage primarily targets the photosystems. In the stroma, several proteins are known to be targets of ROS damage. The inhibitory effects are often ascribed to the oxidation of cysteine residues.

ROS have a strong inhibitory effect on translation in cyanobacteria [348]. The mechanism of the inhibition by H_2_O_2_ is the oxidation of cysteine residues and the subsequent formation of an intramolecular disulfide bond in translation elongation factor G [348], and the formation of a sulfenic acid and an intermolecular disulfide bond in elongation factor Tu [349,350]. The inhibition of translational elongation exerts its effect on the activity of PSII by inhibiting or slowing down the turnover of the D1 protein [351]. Similar ROS effects are expected in chloroplasts.

The Calvin–Benson cycle is inhibited by H_2_O_2_ [352] with the ribulose-1,5-bisphosphate carboxylase oxygenase (rubisco) as the most important target of oxidation [353]. Analysis of the proteome of H_2_O_2_-treated chloroplasts revealed modified cysteine residues in both subunits of rubisco, Fd-dependent glutamate synthase, ferredoxin-NADP^+^ oxidoreductase 1 (FNR1) and glyceraldehyde 3-phosphate dehydrogenase subunit B, and a similar analysis after methyl viologen treatment revealed oxidative changes in 24 chloroplast proteins and modified cysteines in rubisco large subunit, FNR1, myrosinase and NAD(P)-binding Rossman-fold-containing protein [353]. The authors suggested that, due to its large amount, rubisco functions as a redox buffer in the chloroplast.

### 4.5. Damage to Chloroplast DNA

ROS are known to react with DNA [2], and chloroplast DNA is not an exception. A comparison of the integrity of DNA of the chloroplasts of mesophyll and bundle sheath cells of maize, a C4 plant, offers insight into ROS damage within the chloroplasts [354]. In C4 plants, mesophyll cells carry out the photosynthetic electron transfer reactions that produce NADPH and ATP, but also ROS, whereas the bundle sheath chloroplasts are almost devoid of PSII that produces O_2_. A drastically larger amount of DNA damage, analyzed with a long-sequence-specific variant of polymerase chain reaction, was found in the chloroplast DNA of light-grown maize plants in mesophyll cells than in bundle sheath cells [354]. Interestingly, mitochondrial DNA showed a similar difference between mesophyll and bundle sheath mitochondria. Doping soil with Cr(VI) that causes ROS production in leaves also caused damage, visualized by staining with 4′,6-diamidino-2- phenylindole, in the chloroplast DNA [355].

## 5. Detoxification of ROS in Plant Chloroplasts

### 5.1. Detoxification of O_2_^•−^ and H_2_O_2_

Plants have evolved a multitude of enzymatic and non-enzymatic ROS-scavenging and quenching mechanisms. ROS-mediated signaling and ROS detoxification are coupled, as signaling is generally initiated by the oxidation of target molecules, that therefore also act as antioxidants (see reviews [10,356,357]). Here, we discuss the main scavenging mechanisms and antioxidant molecules controlling ROS in the chloroplasts, with emphasis on ROS detoxification in the thylakoid membrane, or stromal-scavenging mechanisms in its immediate vicinity.

O_2_^•−^, produced in chloroplasts, is scavenged efficiently by copper/zinc SODs residing on the stromal face of the thylakoid membrane [12,358]. The dismutation reaction, catalyzed by CuZnSODs, is described in Reactions (5), (44) and (45). While SOD is the main catalyst, the dismutation reaction can also be catalyzed by redox reactive metals such as manganese [359,360], or it can occur non-catalytically [68]. O_2_^•−^ can also oxidize two highly important chloroplast antioxidants, AscH_2_ [62,64] (Reaction (34)) and GSH [66,67] (Reaction (37)).

H_2_O_2_ is reduced by AscH_2_ in a reaction catalyzed by APXs (Reactions (62)–(64)) [93]. The net reaction of H_2_O_2_ scavenging by AscH_2_ can be summarized as (Reaction (126)).

H_2_O_2_ + 2AscH_2_ → 2H_2_O + 2MDA + 2H^+^ (126)

The reaction produces water and MDA. Different APX isoenzymes are found in different chloroplast compartments. Stromal APXs and thylakoid APXs have specific roles in, e.g., plant development, but exhibit functional redundancy in ROS detoxification in mature leaves [361,362,363]. The rest of the ascorbate–glutathione cycle regenerates AscH_2_ [12,364]. The first step is the reduction of MDA to AscH_2_ by Fd_red_ (Reaction (127)),

MDA + Fd_red_ + 2H^+^ → AscH_2_ + Fd (127)

or by NADPH in a reaction catalyzed by MDA reductase (Reaction (128)).

2MDA + NADPH + 3H^+^ → 2AscH_2_ +NADP^+^ (128)

The complete description of the catalytic cycle of reduction of MDA to AscH_2_ is described in [12]. The MDA molecules that are not immediately reduced dismutate non-catalytically, forming AscH_2_ and DHA (Reaction (113)).

GSH donates electrons to DHA either non-catalytically or through catalytic oxidation mediated by DHA reductase, forming AscH_2_ and glutathione disulfide (GSSG) (Reaction (129)).

DHA + 2GSH → AscH_2_ + GSSG (129)

NADPH, formed by the PETC, is then used by glutathione reductase to reduce GSSG back to GSH (Reaction (130))

GSSG + NADPH + H^+^ → NADP^+^ + 2GSH, (130)

thereby completing the ascorbate–glutathione cycle. The functions of APXs in plants have been reviewed by [365]. Because the electrons utilized in the reduction of O_2_ to O_2_^•−^ by PSI originate from water molecules broken down by PSII, and the end product of the production and scavenging of H_2_O_2_ is water, the whole scavenging system is often referred to as the water–water cycle [12].

PRXs, particularly two-cysteine peroxiredoxins (2-Cys PRX), have been shown to function in conjunction with thylakoid APXs in downplaying H_2_O_2_ accumulation during conditions causing oxidative stress in plants [366,367,368]. 2-Cys PRXs facilitate a peroxidative reduction of H_2_O_2_, utilizing electrons from NADPH in a reaction catalyzed by thioredoxin reductase C or, less efficiently, from reduced TRXs [366,367,368]. The catalytic cycle of peroxide detoxification by 2-Cys PRXs and their subsequent regeneration is described in detail in [369]. NADPH is produced both in the light, by PETC, and in the dark, by the oxidative pentose phosphate pathway [370], whereas TRXs are recycled to their reduced form by Fd produced in the light by PSI; the reduction in TRXs is catalyzed by thioredoxin reductases [371]. 2-Cys PRXs are not the only enzymes that can facilitate TRX-dependent H_2_O_2_ detoxification, as glutathione peroxidases also utilize TRXs as substrates instead of reduced glutathiones in plant chloroplasts, and can likely initiate a similar cycle to 2-Cys PRXs [372,373,374,375]. Many other components have been suggested to take part in the recycling of the PRX/glutathione peroxidases-initiated H_2_O_2_ detoxification cycle, such as glutaredoxin, cyclophilins and AscH_2_ [369].

### 5.2. Detoxification of ^1^O_2_

Carotenoids and tocopherols are the main antioxidants against ^1^O_2_ in chloroplasts [43]. Carotenoids function in the NPQ of singlet excited Chl (reviewed in [376,377]), quench ^3^Chl and quench and scavenge ^1^O_2_. Each LHCII subunit contains two luteins, a neoxanthin and a violaxanthin/zeaxanthin [378]. All eight Chl *a* molecules of an LHCII subunit are positioned within close proximity to either of the two luteins or neoxanthin, which facilitates efficient ^3^Chl-quenching especially by lutein, and lowers the probability of ^3^Chl interaction with O_2_, quenching 95% of ^3^Chl in LHCII [43,369,370,371,372,373,374,375,376,377,378,379,380,381]. Violaxanthin and zeaxanthin are not likely to be involved in ^3^Chl quenching in LHCII, as they are bound far from the Chl molecules [382,383]. However, zeaxanthin can quench ^3^Chls in the monomeric Lhcb antenna subunits of PSII (Lhcb4–6) and in the dimeric Lhca subunits of PSI antennae [384]. In LHCII, zeaxanthin is specifically involved in NPQ. In high light, zeaxanthin is produced by violaxanthin de-epoxidase from violaxanthin through the intermediate antheraxanthin, and the newly formed zeaxanthin replaces violaxanthin in LHCII. A switch back to moderate light or darkness induces the epoxidation of zeaxanthin back to violaxanthin and the subsequent replacement of zeaxanthin with violaxanthin in LHCII [385].

The PSII core, consisting of the proximal antennae CP43 and CP47, the Mn-cluster and the RC (D1/D2/Cyt b559) [386,387], binds 11 β-carotenes, two of which are located in the RC [386]. The distance between these two β-carotenes and the RC Chl P680 is too long to allow the participation of the β-carotenes in quenching of ^3^P680 [170,191,386,388]. However, there are indications that β-carotenes in other parts of the isolated PSII core are likely to quench ^3^Chls [386].

The detoxification of ^1^O_2_ itself [192] by carotenoids occurs mainly through physical quenching via electronic energy transfer mechanism (Reaction (17), where A is a carotenoid). The resulting triplet state of the carotenoid (^3^Car) dissipates its excitation energy via a nonradiative transition to its ground state [23,41,43,389]. Carotenoids can also take part in the chemical scavenging of ^1^O_2_ [43,390]. Oxidation products of β-carotene found in plants in high light suggest that ^1^O_2_ can oxidize the β-carotenes of PSII reaction centre [301,390]. β-cyclocitral (β-CC), a volatile product of oxidation of β-carotene by ^1^O_2_, has been shown to be involved in cell signaling [391] (see Section 6.1). In LHCII, ^1^O_2_ produced by the interaction between O_2_ and the residual ^3^Chl that is not quenched by carotenoids, is rapidly inactivated, due to the abundance of carotenoids in LHCII and free carotenoids such as zeaxanthin in the surrounding lipid matrix [388,392].

Other antioxidants in the thylakoid membrane are not bound to LHCs or, in stroma, offer an even greater capacity for the physical or chemical quenching of ^1^O_2_. Tocopherols, or specifically α-tocopherol, are considered as important antioxidants against ^1^O_2_ [393]. The rate constants of the physical quenching of ^1^O_2_ by tocopherols in organic solvents are significantly higher than those of chemical scavenging [389], suggesting that, similarly to carotenoids, the main quenching mechanism by α-tocopherol is physical quenching (Reaction (17)) [43]. However, the oxidation of α-tocopherol by ^1^O_2_ produces 8-hydroperoxy-tocopherone that can be re-reduced to α-tocopherol by AscH_2_ [394,395], which lends the recyclability of the stromal ascorbate-glutathione cycle to ^1^O_2_ detoxification of the lipid phase. AscH_2_ also has the capacity to scavenge ^1^O_2_ (reaction 24) that reaches the stroma [47]. Chloroplasts contain flavonoids in the envelope membrane, and they have the potential to quench ^1^O_2_ both physically and chemically [396,397]. Even though the relatively remote location from the most prominent ^1^O_2_ production sites does put their role as ^1^O_2_ antioxidants in question, flavonoids have been shown to be involved in lowering the amount of ^1^O_2_ in high light in vivo [397]. Other potential ^1^O_2_ antioxidants include polyunsaturated fatty acids [4], PQH_2_ [48,305,306,307,398] and isoprene [399,400].

## 6. ROS Produced by Plant Chloroplasts Function as Signaling Molecules

ROS are known to participate in retrograde signaling, acclimation to biotic or abiotic stresses, programmed cell death (PCD) and many other processes (for recent reviews, see [9,10,11,401,402,403,404,405,406,407]). Here, we aim to briefly summarize what is known (and what is not) about how chloroplast-derived ROS are sensed and how the signaling cascades are initiated. Signaling by ROS produced by enzymes like NADPH-oxidase (reviewed in [408]) will not be discussed here.

### 6.1. Signaling by ^1^O_2_

The lifetime of ^1^O_2_ in plant cells has not been measured, but is generally assumed to be too short (for review, see [23]; Section 2.1.4) to enable diffusion out of chloroplasts and, consequently, ^1^O_2_ itself is unlikely to function as a messenger molecule. Instead, the accumulation of β-CC (a reaction product of β-carotene and ^1^O_2_) has been shown to induce gene expression, leading to stress (e.g., high light) acclimation [391,409,410]. In theory, β-CC could directly travel to the nucleus and activate ^1^O_2_ responsive genes (for discussion, see [411]), however, direct evidence is lacking. Methylene Blue Sensitivity 1 protein might participate in transferring the signal from cytosol to the nucleus [412,413]. In addition, β-CC can be converted to water-soluble β-cyclocitric acid, which also could function as a signaling molecule [410]. Other oxidation products of ^1^O_2_ might have signaling functions, too [346,414].

Another ^1^O_2_-induced pathway involves the Executer1 (EX1) (and possibly Executer2) proteins [415,416]. The oxidation of a tryptophan residue of EX1, presumably by ^1^O_2_ [417], leads to the degradation of EX1 by FtsH, a protease that is also important to the repair cycle of PSII [418]. Afterwards, a signaling cascade leading to PCD is activated [419]. EX1 is not simply a repressor of the PCD pathway [407], however, it is not understood how the degradation of EX1 leads to the induction of PCD. In addition to cell death, EX1 is important in systemic acquired acclimation [420].

The β-CC and EX1 pathways are thought to operate independently [391]. A possible explanation of the need for two pathways is that small amounts of ^1^O_2_ lead to acclimation responses while larger amounts initiate PCD (and still higher amounts cause damage and unregulated cell death [403]). Accordingly, under severe stress, the β-CC pathway, through Oxidative Signal-Inducible 1 kinase, may also lead to PCD, but even this route is EX1-independent [421,422]. Most β-CC is produced from the β-carotene located in the RC of PSII [390,423], and therefore in the grana core [424], whereas EX1 is located in grana margins [425]. As ^1^O_2_ is not expected to diffuse far, the site of production rather than the amount may determine which signaling pathway is activated.

Why do plants need to react differently to ^1^O_2_ produced in different sites? PSII repair occurs mainly in grana margins (for a review, see [426]), and it has been speculated that EX1 would activate PCD if PSII repair is impaired, possibly under adverse environmental conditions when loose Chls might produce ^1^O_2_ [425]. Chl turnover was shown to associate with the repair of PSII [427], implying ^1^O_2_ generation, even though Chl synthesis and degradation are tightly regulated and loose Chls are thought to be bound to specific proteins that prevent ^1^O_2_ production [428]. During a low light to high light transition, the FtsH-protease may get transiently inactivated (possibly indirectly by H_2_O_2_; [429]), thus preventing activation of the EX1-induced PCD. This is in agreement with the view that the EX1 pathway does not respond to high light stress, but it is the β-CC pathway that initiates high light acclimation. Alternatively, the EX1 pathway might be important in plant defense against pathogens [402,430]. ^1^O_2_ produced by Chl catabolites has been proposed to be involved in the hypersensitive response [431], and similarly to the *flu*-mutant [115], ^1^O_2_ produced by Chl catabolites has been suggested to initiate the EX1 pathway also in the wild type [432]. Interestingly, NADPH-protochlorophyllide oxidoreductases were shown to associate with EX1 and FtsH [425], though the interactions may be weak or transient, as they are not always observed [433]. PSII is a target of many pathogens [434] and a non-functional PSII repair cycle might also be involved in plant immunity [435]. However, the physiological role of the EX1 pathway is still unclear.

### 6.2. Signaling by H_2_O_2_

In contrast to ^1^O_2_, the long lifetime of H_2_O_2_ enables its function as a messenger molecule. Exposito-Rodriguez et al. [436] observed that photosynthesis-derived H_2_O_2_ rapidly accumulated in the nuclei, and the addition of cytosolic H_2_O_2_ scavengers did not prevent this. The authors proposed that H_2_O_2_ originated from chloroplasts closely associated with the nucleus. The diffusion of H_2_O_2_ through membranes is not extremely rapid [95], but the transport may be facilitated by (specialized?) aquaporins [95,437,438]. The formation of stromules has been observed under stress [439], and they have been suggested to allow for direct contact between chloroplasts and the nucleus [440]. Another hurdle that chloroplast-originated H_2_O_2_ needs to overcome is that the powerful antioxidant systems of stroma (see Section 5.1) are believed to efficiently scavenge H_2_O_2_. Accordingly, it has been proposed that H_2_O_2_ produced inside the thylakoid membranes (see Section 3.2) might have a great importance in signaling [441]. On the contrary, a meta-analysis of 79 transcriptomic studies concluded that ROS responses are determined by timing rather than the site of origin [442]. Therefore, H_2_O_2_ may participate in multiple pathways, some of which are sensitive to the site of H_2_O_2_ production [443].

H_2_O_2_ is involved in many signaling pathways. For example, photosynthesis-derived ROS, probably H_2_O_2_, may induce enzymatic O_2_^•^^−^ production by cytosolic NADPH-oxidases 408]. In addition, a reduced PQ pool was proposed to cause stomatal closure via H_2_O_2_ accumulation [444]. Borisova-Mubarakshina et al. [445] showed evidence that H_2_O_2_ regulates PSII antenna size in barley during long-term acclimation to high light.

It is not clear what senses H_2_O_2_ in plant cells. SAL1 (an inositol polyphosphate 1-phosphatase) degrades phosphoadenosine phosphate (PAP) in chloroplasts. The oxidation of cysteine residues of SAL1, e.g., under high light, probably by H_2_O_2_, leads to the inactivation of SAL1 and accumulation of PAP [446]. PAP can be transported into the nucleus and activate genes protecting plants from oxidative stress [447,448]. In addition, it has been proposed that a glutathione peroxidase [449], heat shock transcription factors [450], APX [362] and protein phosphatases (reviewed in [451]) might function as H_2_O_2_ sensors.

In general, genes responding to ^1^O_2_ were found to differ from those known to be regulated by H_2_O_2_ [391]. The available data suggest that H_2_O_2_ actually antagonizes EX1-mediated ^1^O_2_ signaling [452,453]. On the other hand, H_2_O_2_ and the β-CC-mediated ^1^O_2_ signaling pathways may converge at Oxidative Signal-Inducible 1 kinase [407,454]. β-CC also down-regulates SAL1 and up-regulates genes generating PAP [391,423], supporting the view that both H_2_O_2_- and β-CC-signaling pathways induce stress acclimation.

### 6.3. Signaling by O_2_^•−^

In plant cells, SOD rapidly converts O_2_^•−^ to H_2_O_2_. The reactivity of O_2_^•−^ may also limit its specificity in signaling. However, the literature suggests that a set of genes is specifically induced by O_2_^•−^ [455,456,457]. For example, Zinc-Finger Protein 12 was shown to be induced more strongly by O_2_^•−^ than by H_2_O_2_ [458].

## Figures and Tables

**Table 1:** Most significant reactive oxygen species (ROS) and ROS derivatives. R is a residual of an organic molecule.

Radicals	Non-Radicals
Reactive oxygen species	
Superoxide anion radical, O_2_^•−^	Singlet oxygen, ^1^O_2_
Hydroperoxyl radical, HO_2_^•^	Hydrogen peroxide, H_2_O_2_
Hydroxyl radical, HO^•^	Ozone, O_3_
ROS derivatives and reactive nitrogen species	
Peroxyl radical, ROO^•^	Organic peroxides, ROOH
Alkoxyl radical, RO^•^	Peroxynitrite ion, ONOO^−^
Nitric Oxide, NO^•^	Alkyl peroxynitrite, ROONO
Nitrogen dioxide, NO_2_^•^	

**Table 2:** Redox midpoint potentials and lifetimes of primary and secondary electron acceptors in PSII. The values were taken from [220,221,222,223,224,225,226,227,228,229,230,231,232,233].

Redox Active Cofactors	Midpoint Redox Potential vs. Normal Hydrogen Electrode (NHE), mV	Lifetime, s	Remarks
Pheo/Pheo^−^	≈−610 [220,221]	(2–5) × 10^−10^ [222]	Reoxidation of Pheo^−^ via forward electron transfer to Q_A_
	−588 [219]		
	−505 [223]	(4–30) × 10^−8^ [222]	Reoxidation of Pheo^−^ via recombination of [P680^+^Pheo^−^]
Q_A_/Q_A_^−^	−80–−200 [224,225]	(0.1–0.2) × 10^−3^ [224,226]	Reoxidation of Q_A_^−^ via forward electron transfer to Q_B_
	Shift from −145 to −70 [227]	(0.3–0.5) × 10^−3^ [224,226,228]	Removal of HCO_3_^−^ bound to acceptor side of PSII.
		(2–4.6) × 10^−3^ [226,228,229]	Reoxidation of Q_A_^−^ via forward electron transfer to Q_B_^−^ and protonation.
		(0.2–2) × 10^−1^ [222]	Reoxidation of Q_A_^−^ by PQ that binds to an empty Q_B_ site.
		1–2 [226]	Reoxidation of Q_A_^−^ via charge recombination with oxidized TyrZ.
			Reoxidation of Q_A_^−^ via charge recombination with S_2_
Q_A_^−^/Q_A_^2−^	−500 [230]		Double reduction achieved either by chemical treatment or by strong illumination in anaerobic conditions. No doubly reduced Q_A_ accumulates during aerobic light treatment.
Q_B_/Q_B_^−^	−45–−60 [220,231,232]	(0.3–0.5) × 10^−3^ [224,226,228]	Reduction of Q_B_^−^ via electron transfer from Q_A_^−^
		>0.4 [222]	Reoxidation of Q_B_^−^ via charge recombination with oxidized TyrZ.
		30 [222]	Reoxidation of Q_B_^−^ via charge recombination with S_2_
Q_B_/Q_B_H	100 [220]	(0.3–0.5) × 10^−3^ [224,226,228]	Assuming the same time as for Q_B_^−^
		>0.4 [222]	
		30 [222]	
Q_B_^−^/Q_B_^2−^	−200–−464 [233]		
Q_B_H/Q_B_H^−^	290–373 [233]		

## Abbreviations

β-CC	β-cyclocitral
^1^Chl, ^1^Chl* and ^3^Chl	respectively, singlet state, singlet excited state and triplet excited state of chlorophyll
^1^O_2_	singlet oxygen (^1^∆gO_2_)
2-Cys PRX	two-cysteine peroxiredoxin
A	acceptor
A_1_	phylloquinone of PSI
APX	ascorbate peroxidase
AscH_2_	ascorbate, ascorbic acid
Car and ^3^Car	respectively, singlet and triplet state of carotenoid
CAT	catalase
Chl and Chl*	respectively, chlorophyll and excited chlorophyll
Chl *a*	chlorophyll *a*
cyt	cytochrome
cyt b559	cytochrome b559
Cyt b6f	cytochrome b6/f complex
DBMIB	2,5-dibromo-6-isopropyl-3-methyl-1,4-benzoquinone
DCMU	3-(3,4-di-chlorophenyl)-1,1-dimethyl urea
DHA	dehydroascorbate; DMF, dimethylformamide
DNP-INT	2-(2,4-dinitrophenoxy)-3-iodo-4-methyl-1-(1-methylethyl)-5-nitro-benzene
*Em*	midpoint redox potential
*E*_0_′	standard redox potential
EPR	electron paramagnetic resonance
EX1	Executer1
Fd, Fd_ox_ and Fd_red_	ferredoxin, and oxidized and reduced ferredoxin, respectively
FL	flavin
FL^•−^	anion form of flavin semiquinone
FLH^•^	flavin semiquinone radical
FLHOOH	flavin hydroperoxide
FLU	a protein important in control of chlorophyll biosynthesis
FNR	ferredoxin-NADP^+^ reductase
FtsH	a protease involved in PSII repair; F_X_, F_A_, and F_B_, 4Fe-4S clusters of PSI
GSH	reduced glutathione
GSSG	glutathione disulfide
H_2_O_2_	hydrogen peroxide
HO^•^	hydroxyl radical
HO_2_^•^	hydroperoxyl radical
HO_2_^−^	hydroperoxyl anion
HP, IP, LP and VLP	respectively, high, intermediate, low and very low potential forms of cytochrome b559
hν	energy of a photon
ISC	intersystem crossing
*k_forward_* and *k_reverse_*^•^	forward and reverse rate constant, respectively
LHC	light harvesting complex; LHCI and LHCII, respectively, light harvesting complex of PSI and PSII
LOO^•^	lipid peroxyl radical; M, metal
MDA	monodehydroascorbate
MDAR	monodehydroascorbate reductase
MenB	1,4-hydroxynaphthoyl-coenzyme A synthase
NHE	Normal Hydrogen Electrode
NPQ	non-photochemical quenching of excitation energy
O_2_	molecular oxygen (^3^Σ^+^_g_O_2_); O_3_, ozone
O_2_^•−^	superoxide anion radical
OEC	oxygen-evolving complex
P680	primary donor of PSII
P700	primary donor of PSI
PAP	phosphoadenosine phosphate
PC	plastocyanin
PCD	programmed cell death
PETC	photosynthetic electron transport chain
Pheo	pheophytin
Pheo_D1_ and Pheo_D2_	respectively, pheophytins bound to D1 and D2 proteins of PSII
PPFD	photosynthetic photon flux density
PQ	plastoquinone
PQ^•−^	plastosemiquinone anion radical
PQH_2_	plastoquinol
PRX	peroxiredoxin; PSI and PSII, Photosystems I and II, respectively
PsbS	a chloroplast-localized protein required for NPQ
PTOX	plastid terminal oxidase
PX	peroxidase
Q	quinone
Q^•−^	semiquinone anion radical
R^•^	organic radical
RC	reaction center
ROO^•^	peroxyl radical
ROOH	organic peroxide
ROOOOR	linear tetraoxide
ROS	reactive oxygen species
RS	thiol
rubisco	ribulose-1,5-bisphosphate carboxylase oxygenase
S	sensitizer
SAL1	an inositol polyphosphate phosphatase
SOD	superoxide dismutase
TRX	thioredoxin
TyrZ	the redox active tyrosine of PSII
